# Screening of cellulolytic bacteria from rotten wood of Qinling (China) for biomass degradation and cloning of cellulases from *Bacillus methylotrophicus*

**DOI:** 10.1186/s12896-019-0593-8

**Published:** 2020-01-07

**Authors:** Lingling Ma, Yingying Lu, Hong Yan, Xin Wang, Yanglei Yi, Yuanyuan Shan, Bianfang Liu, Yuan Zhou, Xin Lü

**Affiliations:** Lab of Bioresources, College of Food Science and Engineering, Northwest A&F University, Yangling, 712100 Shaanxi Province China

**Keywords:** Rotten wood, *Bacillus methylotrophicus*, Cellulase, Biomass, Cloning and expression

## Abstract

**Background:**

Cellulosic biomass degradation still needs to be paid more attentions as bioenergy is the most likely to replace fossil energy in the future, and more evaluable cellulolytic bacteria isolation will lay a foundation for this filed. Qinling Mountains have unique biodiversity, acting as promising source of cellulose-degrading bacteria exhibiting noteworthy properties. Therefore, the aim of this work was to find potential cellulolytic bacteria and verify the possibility of the cloning of cellulases from the selected powerful bacteria.

**Results:**

In present study, 55 potential cellulolytic bacteria were screened and identified from the rotten wood of Qinling Mountains. Based on the investigation of cellulase activities and degradation effect on different cellulose substrates, *Bacillus methylotrophicus* 1EJ7, *Bacillus subtilis* 1AJ3 and *Bacillus subtilis* 3BJ4 were further applied to hydrolyze wheat straw, corn stover and switchgrass, and the results suggested that *B. methylotrophicus* 1EJ7 was the most preponderant bacterium, and which also indicated that *Bacillus* was the main cellulolytic bacteria in rotten wood. Furthermore, scanning electron microscopy (SEM) and X-ray diffraction analysis of micromorphology and crystallinity of wheat straw also verified the significant hydrolyzation. With ascertaining the target sequence of cellulase β-glucosidase (243 aa) and endoglucanase (499 aa) were successfully heterogeneously cloned and expressed from *B. methylotrophicus* 1EJ7, and which performed a good effect on cellulose degradation with enzyme activity of 1670.15 ± 18.94 U/mL and 0.130 ± 0.002 U/mL, respectively. In addition, based on analysis of amino acid sequence, it found that β-glucosidase were belonged to GH16 family, and endoglucanase was composed of GH5 family catalytic domain and a carbohydrate-binding module of CBM3 family.

**Conclusions:**

Based on the screening, identification and cellulose degradation effect evaluation of cellulolytic bacteria from rotten wood of Qinling Mountains, it found that *Bacillus* were the predominant species among the isolated strains, and *B. methylotrophicus* 1EJ7 performed best on cellulose degradation. Meanwhile, the β-glucosidase and endoglucanase were successfully cloned and expressed from *B. methylotrophicus* for the first time, which provided new materials of both strain and the recombinant enzymes for the study of cellulose degradation and its application in industry.

## Background

Cellulosic biomass (composed of cellulose, lignin and hemicellulose) is one of the most abundant renewable resources, which is also considered as a potential and promising raw material for future energy production [1]. Cellulose has been reported as the critical component that can be converted into various value-added products: e.g. ethanol, 5-hydroxymethylfurfural (HMF), levulinic acid, butanol, alkanes, hexane, succinic acid, ethyl lactate, and other chemicals [2–4], and no matter which procedure conducted, cellulose should be firstly hydrolyzed to glucose, and then various bio- or chemical processes can be really carried out. Therefore, the degradation of cellulosic material has attracted huge attentions for the improvement of the efficiency and cleaning process.

There were various methods proposed to hydrolyze cellulose, including acid-activated montmorillonite catalysts, steam explosion, acid, alkaline, enzymatic hydrolysis and microbiological methods [5–7]. In view of protecting of environment and saving energy, the enzymatic and microbiological methods were prioritized to be practically applied, and what’s more, both of which were associated with microorganisms, such as fungi and bacteria [5]. Indeed, fungi exhibit a strong ability to secret considerable extracellular enzymes including multi-cellulases, which was the main reason why numerous studies had been conducted on fungi producing cellulases, such as *Trichoderma reesei* RUT-C30 [8], *Trichoderma koningiopsis* FCD3–1 [9], and *Melanoporia* sp. CCT 7736 [10]. However, it has also been found that the culture and genetically modification of fungi were relatively more difficult to achieve than bacteria, which seriously hindered the practical application of fungi and fungi-producing cellulases to celluloses hydrolyzation [11, 12]. In general, bacteria were commonly considered as a powerful tool for functional modification or genomic operation, for instance, the heterogeneous cloning and expressing of single cellulase or recombinant cellulases. Unfortunately, the library of bacteria that possessed powerful activity to hydrolyze cellulose was not sufficient, which partly limited the study and application of the cellulolytic bacteria. Therefore, lots of researches about screening of cellulolytic bacteria had been conducted and reported, such as *Bacillus* sp. BS-5 [13], *Bacillus licheniformis* 2D55 [14], *Bacillus subtilis* BY-4 [15], *Paenibacillus chitinolyticus* CKS1 [16], *Ochrobactrum sp* K38 [17], and *Clostridium thermocellum* [18], which also suggested that various species of bacteria from different origins should be screened and highlighted.

The Qinling Mountains (32°30′N-34°45′N and 104°30′E-112°45′E) are located in the center of China and has 1500 km in length, which act as a crucial geographic demarcation line separating semi-arid area and humid regions in China [19]. It is well known that Qinling Mountains possess the unique climate, plants, and microorganism resource. Hence, rotten woods originating from Qinling Mountains contains various of biomass degrading microorganisms, which provides good materials for the screening of valuable bacteria for lignocellulose degradation. Consequently, in the present study, bacteria with the capability of cellulose degradation were screened and identified from rotten woods of Qinling Mountains. Subsequently, cellulase activities were assayed and the strains were inoculated with the wheat straw, corn stover and switchgrass to investigate the degradation effect on lignocellulosic biomass. Furthermore, for the purpose to verify the possibility of the heterologous expression for the cellulase from *B. methylotrophicus* 1EJ7, the cloning and expression of the proposed enzymes were conducted. Based on the target sequence exploring in National Center for Biotechnology Information (NCBI) database, β-glucosidase and endoglucanase with food cellulase activity were successfully cloned and expressed on the pET-28a(+) plasmid in *E.coli* BL21 (DE3), which provide valuable materials for the further study about cellulase in engineering modifications and application to industry.

## Results

### Isolation and identification of cellulolytic bacteria

A total of 81 strains were isolated from five rotten wood samples, in which 8, 17, 19, 15 and 22 isolates were obtained from weed tree, red birch, poplar, alpine rhododendron and willow, respectively. Meanwhile, based on “diameters ratio between clear zone and strain” during the investigation by Congo red method (Additional file 1: Figure S1) and the growth of strains in the process of subculture, 55 cellulolytic strains were finally selected for the further study. In addition, it needed to be mentioned that strains named as *B. subtilis* 1CJ1 and *Bacillus* sp. 1CJ4 had the largest diameters of clear zone more than 25 mm, and the largest value of “diameters ratio between clear zone and strain” was 3.71 belonged to *Bacillus sp.* 3AJ7 (Additional file 2: Table S2).

**Fig. 1:**
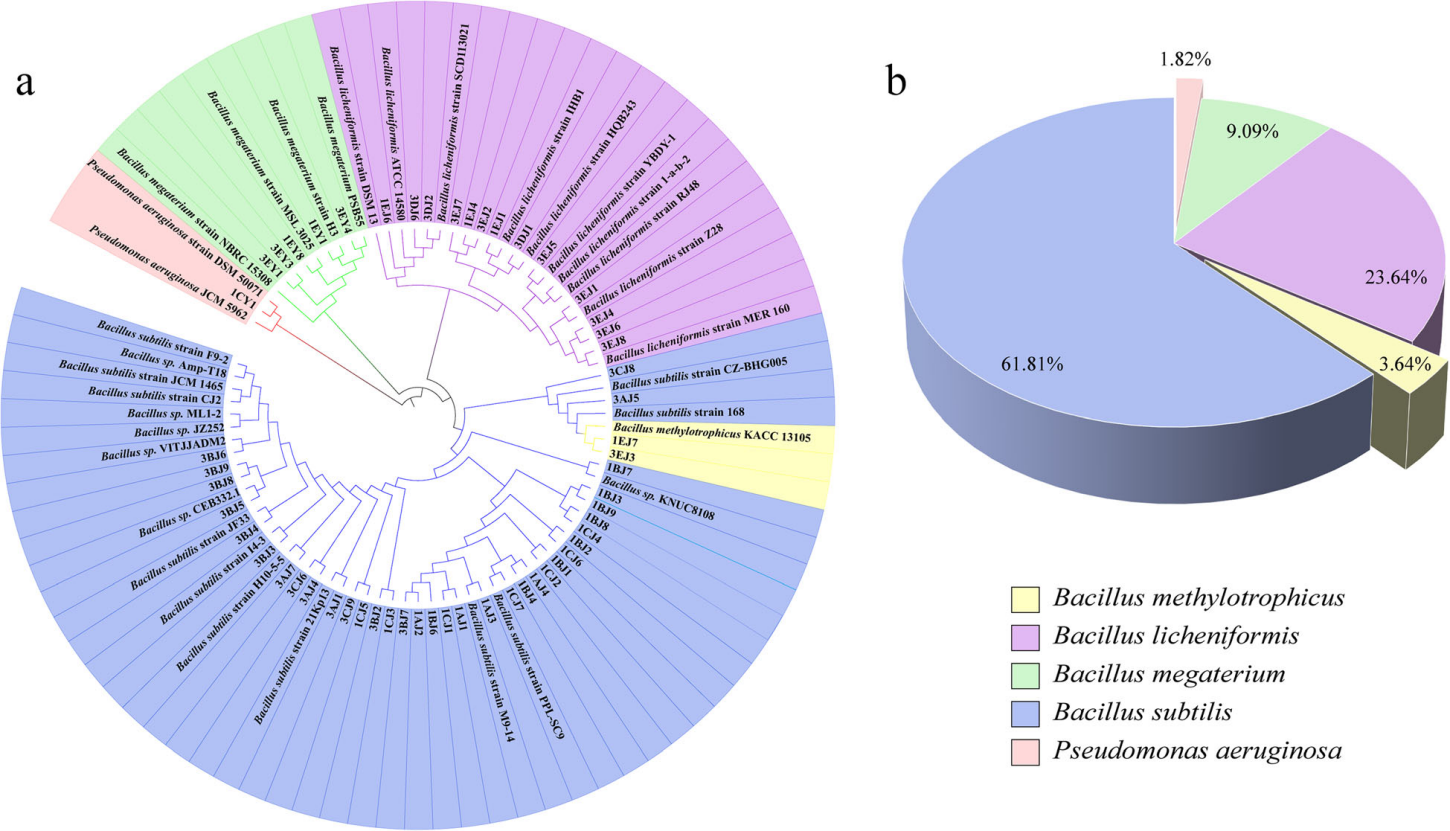
Phylogenetic tree and strains distribution of cellulosic bacteria. **a** Circular maximum likelihood phylogenetic tree of bacterial of 16S rDNA sequence. The tree was constructed in iTOL. **b** Strains distribution. **a** and **b** with the same color band of different species

The isolated strains were identified according to their 16S rRNA gene, after which phylogenetic tree was established as shown in Fig. 1. Results indicated that the strains could be classified into *Bacillus subtilis*, *Bacillus* sp., *Pseudomonas aeruginosa*, *Bacillus licheniformis*, *Bacillus methylotrophicus* and *Bacillus megaterium*, which suggested that the *Bacillus* might be the predominant strains possessing the cellulose degradation activity in the rotten wood.

### Cellulase activities and hydrolysis capability

**Fig. 2:**
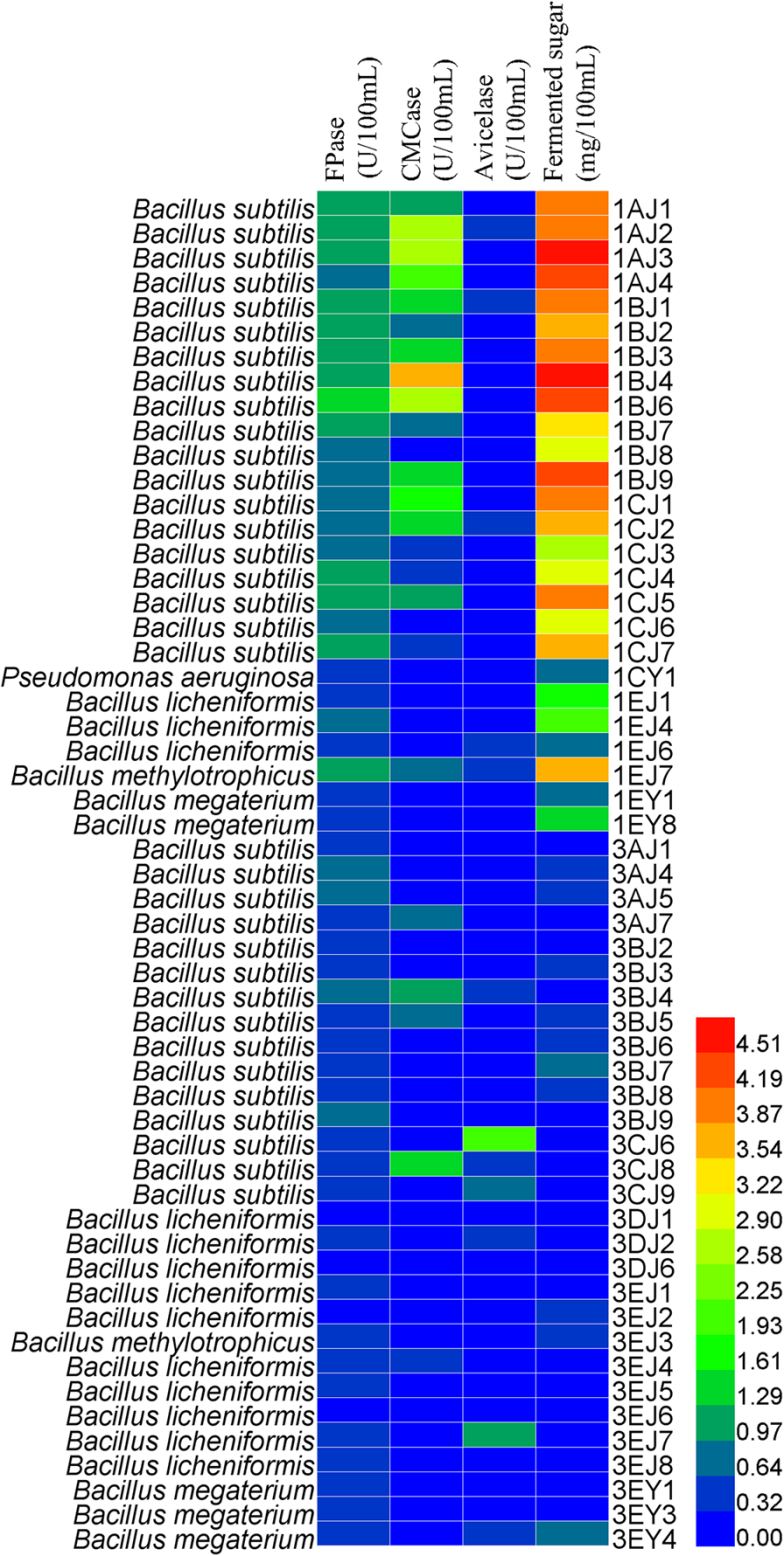
Heat map of reducing sugar production and enzyme activities

The isolated strains were inoculated into sole carbon source medium for 48 h at 37 °C under 120 rpm. Reducing sugar concentration and cellulase activities were investigated and shown in Fig. 2 as a heat map which obviously indicated the relationship between bacteria and the cellulase activity as well as the production of cellulose degradation (reducing sugar content). In addition, the detail results were supplied in the Additional file 3: Table S3.

The crude enzyme extracts were collected to determine reducing sugar content and cellulase activities. The maximum reducing sugar concentration was observed in CMC-Na medium by *B. subtilis* 1AJ3 of 4.83 mg/100 mL, followed by *B. subtilis* 1BJ4 of 4.54 mg/100 mL and *B. subtilis* 1BJ6 of 4.47 mg/100 mL. Compared with CMC-Na medium, a maximum production of reducing sugar content (1.61 mg/100 mL) was obtained by *B. subtilis* 3BJ7 in Avicel medium. The results showed that strains selected from CMC-Na medium had a higher enzyme activity than that selected from Avicel. In addition, *B. subtilis* 1BJ4 had both the highest FPase activity (0.0133 U/mL) and CMCase activity (0.0368 U/mL), while *B. licheniformis* 3EJ7 had the highest Avicelase activity of 0.010 U/mL. For the majority of strains, the results were coincided with the general sense that strains selected from CMC-Na always had a high CMCase activity, while that from Avicel had a high Avicelase activity. It was also interesting to find that strains processing both CMCase activities and Avicelase activities did not appear in present study. For example, *B. subtilis* 1BJ4 had the highest CMCase activity, but it didn’t exhibit Avicelase activity, which was possibly explained by the fact that different strains might produce different cellulases under the same or different carbon source [20]. Interestingly, although *B. subtilis* 3CJ6 had the highest Avicelase activity, and both other two enzyme activities were tested, no reducing sugar was detected.

From the heat map, it found that the difference of intergeneric impacted the cultivation process and which led to the differences in reducing sugar production and cellulase activities. As the results shown, *B. subtilis* strains possessed the advantages in secreting of cellulases, which led to the high reducing sugar content, FPase and CMCase activity. Meanwhile, *B. methylotrophicus* and *B. licheniformis* performed relatively well just in the reducing sugar content. In addition, bacteria isolated from CMC-Na as solo carbon source medium had the higher FPase and CMCase activities than that from Avicel medium.

According to the reducing sugar content and cellulase activities, eight strains were further selected as *B. subtilis* 1AJ2, *B. subtilis* 1AJ3, *B. subtilis* 1BJ4, *B. methylotrophicus* 1EJ7, *B. subtilis* 3BJ4, *B. subtilis* 3CJ6, *B. subtilis* 3CJ8 *and B. methylotrophicus* 3EJ7, which would be applied to carbon sources to evaluate the cellulose degradation activity.

### Reducing sugar production and cellulase activities in different carbon sources

**Fig. 3:**
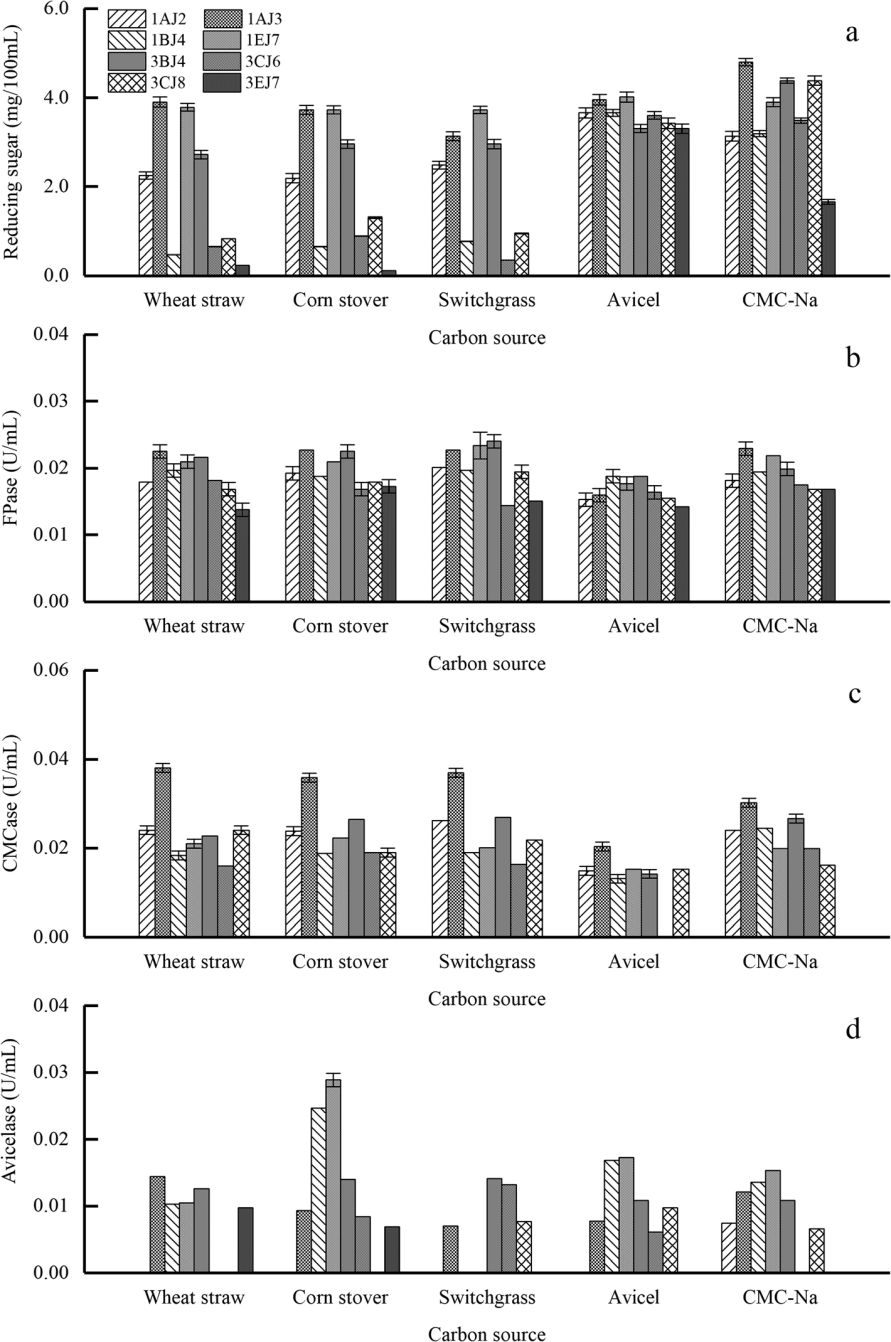
Reducing sugar and cellulase activities of eight strains in different carbon source medium. **a** Reducing sugar content; **b** FPase; **c** CMCase; **d** Avicelase

The selected eight strains were cultured with different carbon sources: wheat straw, corn stover, switchgrass, Avicel and CMC-Na (Fig. 3).

Each strain was separately inoculated into the medium with five different carbon sources (wheat straw, corn stover, switchgrass, Avicel, and CMC-Na) for 48 h with 6% seed inoculation. Figure 3a shows the reducing sugar concentration obtained from different carbon sources by the treatment with each strain. *B. subtilis* 1AJ3 and *B. methylotrophicus* 1EJ7 showed strong potential in the producing of reducing sugar, even in the lignocellulosic biomass without any other pretreatments (wheat straw, corn stover, and switchgrass), which then followed by *B. subtilis* 3BJ4 and *B. subtilis* 1AJ2. It also found that the strains showed similar FPase and CMCase activities (Fig. 3b and Fig. 3c) in different carbon sources. Specifically, only *B. subtilis* 1AJ3 and *B. subtilis* 3BJ4 produced Avicelase in all medium, and *B. subtilis* 1AJ2 only produced Avicelase in CMC-Na medium. Meanwhile, other strains produced Avicelase in three or more carbon sources. Accordingly, based on the reducing sugar content, cellulase activities and carbon source type, three strains (*B. subtilis* 1AJ3, *B. methylotrophicus* 1EJ7, and *B. subtilis* 3BJ4) were selected for the further study.

### Pretreatment of lignocellulosic biomass

Three strains of *B. subtilis* 1AJ3, *B. methylotrophicus* 1EJ7 and *B. subtilis* 3BJ4 were used to pretreat wheat straw, switchgrass and corn stover separately or mixed-up. After sterilization at 121 °C for 20 min, the initial reducing sugars concentration were 136.34 mg/100 mL, 109.46 mg/100 mL, and 39.16 mg/100 mL in the medium of corn stover, switchgrass and wheat straw, respectively.

**Fig. 4:**
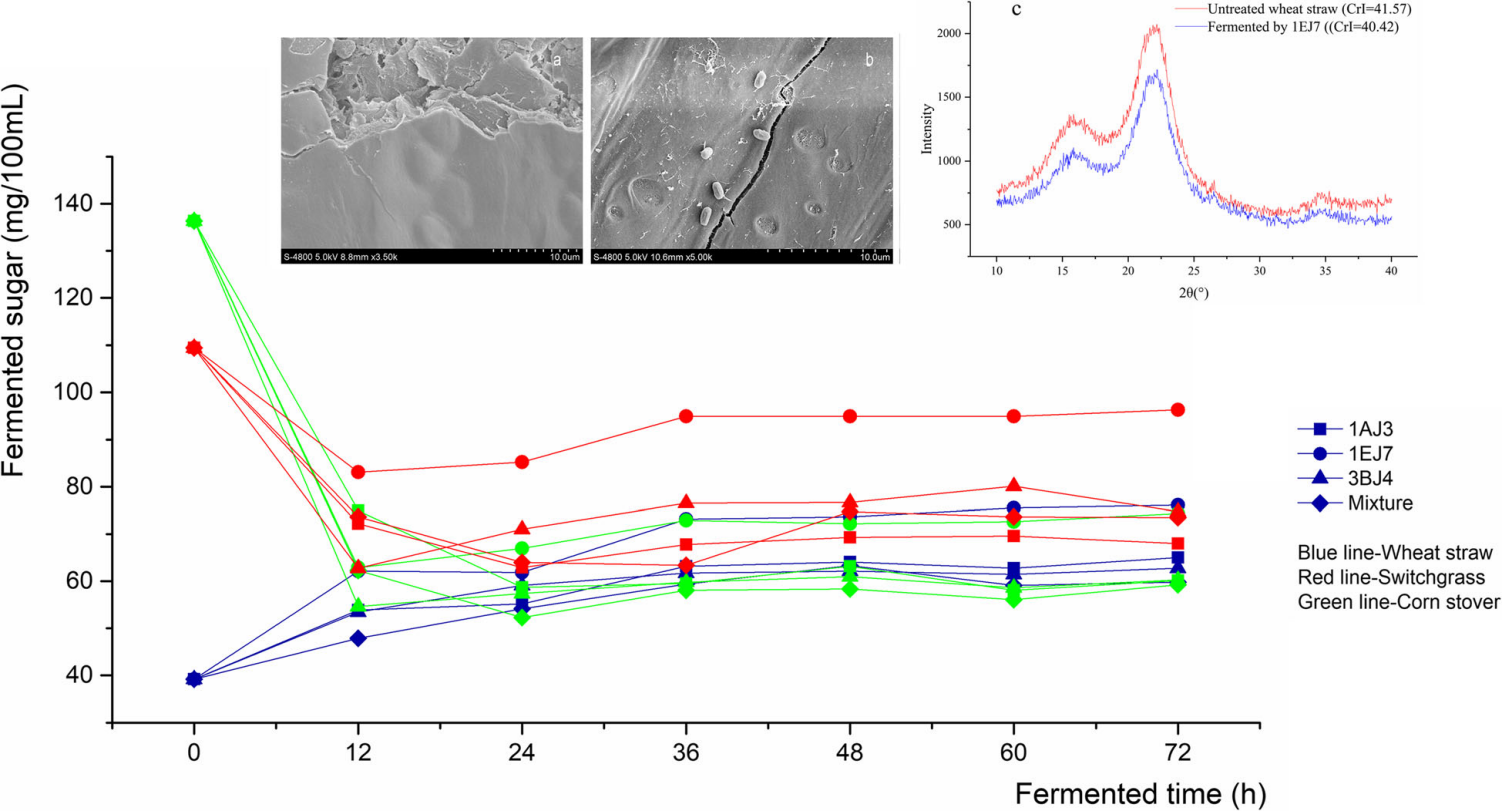
Reducing sugar production by different strains in 7% wheat straw, corn stover and switchgrass for 72 h. SEM of wheat straw before (**a**) and after (**b**) 72 h fermentation by *B. methylotrophicus* 1EJ7. X-ray of untreated wheat straw and fermented by *B. methylotrophicus* 1EJ7 was showed in **c**

The reducing sugar content in all samples tended to be stable (Fig. 4) after culturing 36 h, and the highest sugar content of 95 mg/100 mL was obtained by *B. methylotrophicus* 1EJ7 in switchgrass. Meanwhile, 73 mg/100 mL in wheat straw and 72 mg/mL in corn stover were also obtained by *B. methylotrophicus* 1EJ7, which also indicated that no synergistic effect was observed in the pretreatment of the mixture.

SEM test was benefit for understanding the process of the straw degradation caused by the proposed strains. As one of the major agricultural waste in China, wheat straw has a relatively denser lignocellulosic structure, and which was selected as the sample to be investigated after the hydrolyzation by *B. methylotrophicus* 1EJ7. It was found that the surface (Fig. 4a) of the wheat straw particles was dramatically changed (Fig. 4b) after bacteria pretreatment. Specifically, it was obviously found that the smooth surface of wheat straw particles was destroyed to form numerous holes and lots of bacteria were observed as adhering on the surface. Therefore, the sunken tiny holes suggested that the bacteria processed the cellulase activity and which initially destroyed the surface structure of wheat straw. In addition, the similar phenomenon was also observed in corn stover hydrolysis [21].

As the cellulose content affected the degree of crystallinity in various of plant biomass, the decrease of crystallinity is also an index of the decrease of cellulose content or the destruction of the cellulose structure, therefore, which was also used to evaluate the efficiency of the pretreatment [22]. Hence, X-ray diffraction was used to analyze crystallinity of wheat straw samples in the present study. The results indicated that the Cr *I* of wheat straw decreased from 41.57 to 40.52 (Fig. 4c) after the pretreatment, which also verified the degradation of wheat straw caused by the pretreatment of *B. methylotrophicus* 1EJ7.

### Cellulases clone and expression

**Fig. 5:**
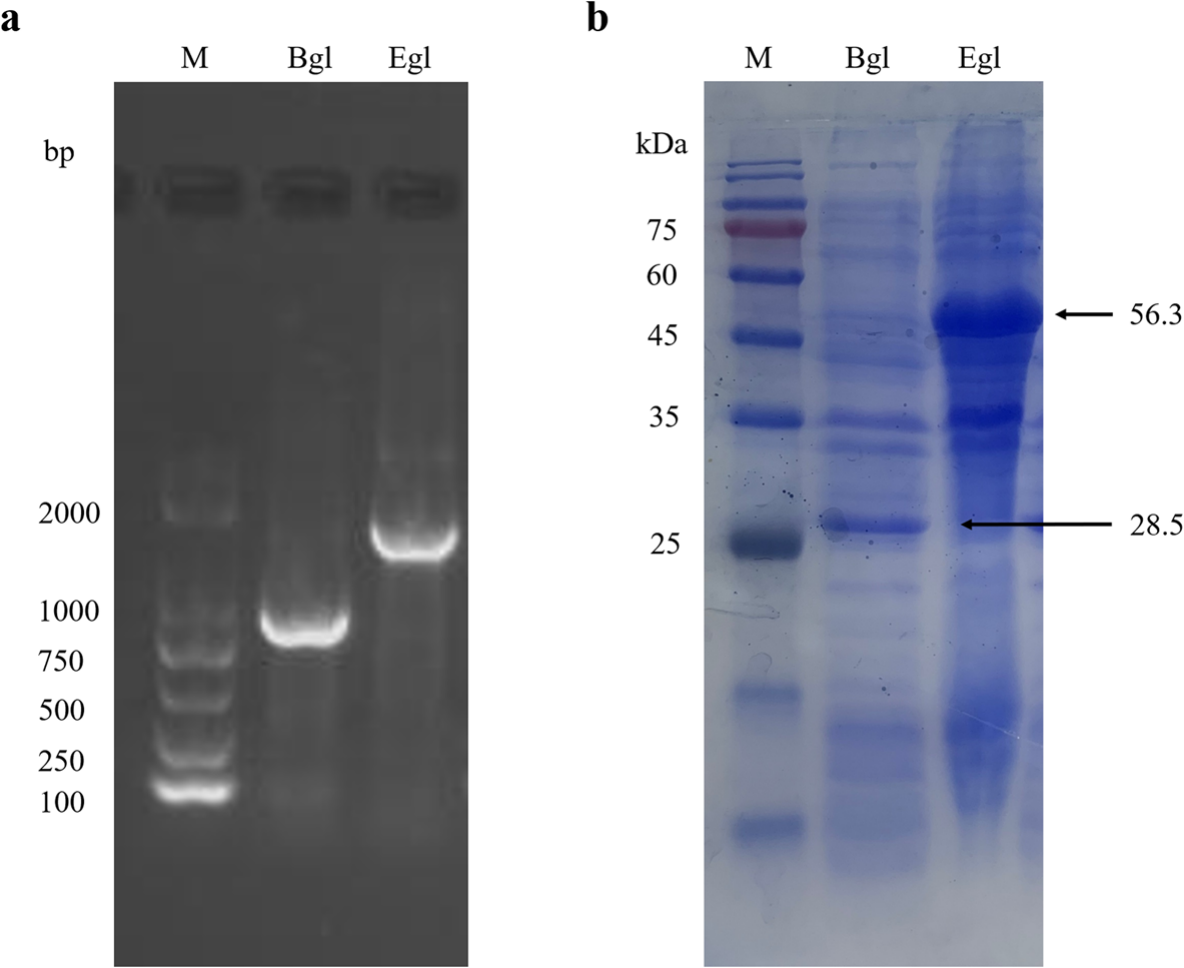
Agarose gel electrophoresis and SDS-PAGE of Bgl and Egl. **a** 1% Agarose gel electrophoresis use universal primer of T7/T7er by the recombined plasmid. **b** 15% SDS-PAGE for expression of Bgl and Egl

The target genes of β-glucosidase and endoglucanase were 732 bp and 1500 bp, respectively, and which were successfully cloned. In addition, universal primer T7/T7er was utilized to amplify the two recombinant plasmids (pET-28a-Bgl and pET-28a-Egl), and then PCR products were tested for the complete sequences. Thereafter, heterologous expressions of the proposed two genes in *E. coli* BL21 (DE3) were carried out to obtain the enzymes. As shown in SDS-PAGE, two cellulases were both successfully expressed in *E.coli* BL21 (DE3), and the *Mw*s were tested as 28.5 kDa and 56.3 kDa (Fig. 5), respectively. Bgl and Egl crude cellulase activities were 1670.15 ± 18.94 U/mL and 0.130 ± 0.002 U/mL, respectively (Additional file 4: Table S4).

The results of domains analysis showed that Bgl belonged to GH16 family (Additional file 5: Figure S5), and Egl contained two domains as catalytic domain (CD) and carbohydrate-binding module (CBM), which belonged to GH5 family and CBM3 family, respectively (Additional file 6: Figure S6).

Meanwhile, by blast on PDB (protein database) website, the highest identification of Bgl was endo-beta-1,3–1,4 glucanase (PDB id 3O5S_A) from *Bacillus subtilis* 168 with a similarity of 93.55%, and Egl was 94.92% similarity with endo-1,4-beta-glucanase (PDB id 3PZT_A) and 90.41% with CBM3 lacking the calcium-binding site (PDB id 2L8A_A) from *B. subtilis* 168. In addition, compared with Bgl sequence of *Bacillus velezensis* JTYP2, it was found that only four amino acids (70 M → V, 96 V → A, 156A → K, 204 N → T) were different with the Bgl in present study, and the predicted secondary structure didn’t obviously affect by these differences. It also indicated the Bgl of *B. subtilis* 168 showed more differences with the proposed Bgl as 22 amino acids were different (Additional file 7: Figure S7). For the Egl, the sequence analysis showed that it had a 96.6% similarity with the Egl of *Bacillus velezensis* JTYP2, and there are 51 different bases between the two sequences led to 17 different amino acids (27A → T, 31G → E, 52Q → R, 199P → I, 238S → F, 285 K → N, 316S → T, 331S → G, 332 N → T, 334S → L, 339A → G, 364S → R, 382 T → A, 404F → V, 411I → M, 414S → G, 440 K → T), in which most changes appeared on the carbohydrate-binding module (CBM) and the linked peptide [23], and all the changes of the amino acids didn’t significantly influent the secondary structure (Additional file 8: Figure S8).

### Bioinformatics analysis and homology modeling

The recombined Bgl contains 251 amino acids included a His-tag with a molecular weight of 28.47 kDa. The computed pI was 6.79, and the negative GRAVY score (− 0.491) suggested that the protein might be hydrophilic. Bgl showed instability index and aliphatic index of 16.14 and 60.24, respectively. Correspondingly, the recombined Egl contained 507 amino acids including his-tag with a predicted molecular weight of 56.32 kDa, and the computed pI was 7.26 and the negative GRAVY score (− 0.616) suggested the Egl was a hydrophilic protein. In addition, Egl showed instability index and aliphatic index of 29.60 and 73.69, respectively, and instability index less than 40 also indicated that both the Bgl and Egl from *B. methylotrophicus* 1EJ7 was stable.

**Fig. 6:**
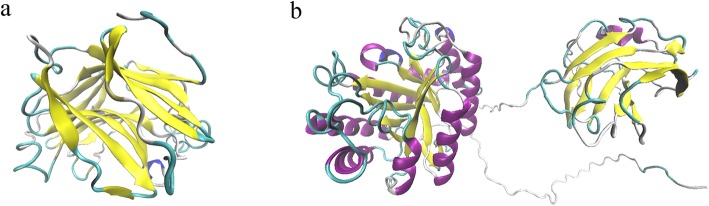
3D structure prediction of Bgl (**a**) and Egl (**b**)

Bgl (Fig. 6a) and Egl (Fig. 6b) homology structural models were obtained by the I-TASSER. The information about the active site was obtained through superimposing 3D model structure of the Bgl with the template structure of cellulase from *Paenibacillus macerans* hybrid endo-1,3-1,4-beta-D-glucan 4-glucanohydrolase (PDB id 2AYH) [24], which provided accuracy of homology between two structures and also contributed to find the conserved active site residues. Active sites of Bgl were represented by 6 amino acid residues as Leu103, Phe105, Thr175, Asp179, Tyr188 and Asp236. The 35th to 242th amino acid domain of Bgl included a classical sandwich-like beta-jelly roll fold, and formed by two main, closely packed and curved antiparallel beta sheets, which led to a deep channel harboring the catalytic machinery. Bgl was found to be a catalytic sequence motif similar with GH16 family [23, 25], E-[ILV]-D-[IVAF]-[VILMF] (0, 1)-E, which was formed by amino acid 134th to 138th (EIDIE). The structural analysis of Egl showed that domains of CD and CBM, in which the catalytic domain had the critical TIM-barrel fold structure of GH5 family, consisting of 8 β-strands surrounded by 8 α-helices with the active site located at the cleft.

## Discussions

### The unique cellulolytic bacteria in rotten woods from the Qinling Mountains

Microbial biodegradation has been applied as an environmental-friendly method in cellulosic materials to produce various valuable compounds. Over the past decades, numerous cellulolytic microorganisms have been screened and characterized. In this study, it was found that the strains isolated from Qinling rotten wood distributed in a widely taxonomic coverage (e.g. *Bacillus subtilis, Pseudomonas aeruginosa, Bacillus licheniformis, Bacillus methylotrophicus and Bacillus megaterium*), in which *Bacillus subtilis* was found as the most abundant species.

Similar with the related studies, Bacillus strains were the dominant bacteria in rotten wood during the process of screening by LB as enrich medium, which might attribute to the robust enzymes secretory system of the *B. subtilis* strains [26, 27]. Meanwhile, this results also suggested that *B. subtilis* was a promising resource of potential cellulase enzymes. Besides, *B. subtilis* was also detected in forest system, such as forest soil [28], freshwater swamp forest [29]. In addition, *B. licheniformis* was another vital species found in rooden trees, exhibiting the ability to degrade cellulose or other natural cellulosic biomass, and which has been extensively found in hot spring, soil, gut of animals, paddy field and agricultural environment, with a high production of cellulase [30, 31]. It is noteworthy that *B. megaterium, B. methylotrophicus* and *Pseudomonas aeruginosa* in this study were scarcely reported to exhibit the ability to produce cellulase or degradation activity of cellulosic materials. To the best of our knowledge, only *B. megaterium* BM05 has been reported for wheat straw fermentation [32], and *B. methylotrophicus* Y37 [33] were reported to have cellulolytic enzyme. Accordingly, it suggested that the strains screened in present study possessed the special bacteria composition for novel cellulases development. Meanwhile, various species of cellulolytic bacteria were also identified, such as *B. subtilis, P. aeruginosa, B. licheniformis, B. methylotrophicus* and *B. megaterium*, which have been rarely reported. Consequently, the results revealed that the special areas (e.g. Qinling mountains) were good sources for the development of cellulolytic bacterium, and the *B. subtilis* might need to be paid more attentions during the potential bacterial screening.

### Hydrolysis capability of the strains and its cellulolytic enzymes

On the whole, wild bacteria exhibit low enzyme production capacity and generally low enzyme activity. In this study, CMC-Na medium with different stains had the maximum reducing sugar content of 4.83 mg/100 mL, while Avicel medium only had 1.61 mg/100 mL reducing content (Fig. 2), revealing that the hydrolysis capability of stains can be affected by the types of cellulose substrates. Moreover, the sugar consumption for the strain’s growth was also a cause of the low content of reduced sugar in cultivation broth. It was also reported in some studies that no reducing sugars were detected in the finial CMC medium when cultured with the isolated bacteria [34].

The strains of *B. subtilis* 1AJ3, *B. methylotrophicus* 1EJ7 and *B. subtilis* 3BJ4 all belonged to *Bacillus*, which were selected and applied to the pretreatment of switchgrass for their relatively higher cellulase activity and better cellulose degradation efficiency. Although the three strains all processed the cellulose hydrolyzation activity in various types of substrates, *B. methylotrophicus* 1EJ7 performed best for the highest reducing sugar content in the final broth compared with other two strains. It was interesting to find that Avicelase activity was only not detected in *B. methylotrophicus* 1EJ7 in the medium with switchgrass as only carbon source, which was mainly attributed to the different enzyme system of the strains. In detail, although the *B. subtilis* has the powerful ability for the enzyme secretion, Avicelase needed special carbon sources to induce in the *B. methylotrophicus* 1EJ7, and as the switchgrass was not the suitable carbon sources for the *B. methylotrophicus* 1EJ7, which led to the lacking of Avicelase activity. In addition, as an herbaceous plant resource, switchgrass was easily to be degraded compared to wheat straw and corn stover, therefore, it was used for the effect investigation of the biomass degradation caused by the proposed strains. What’s more, when the pretreatment with the strains in a high concentration of substrates, the reducing sugar content in the final broth was the most important index to evaluate the pretreatment efficiency, since the high reducing sugar content was the preconditions for the conversion of ethanol and other valuable chemical products. In this view, compared with other two strains, *B. methylotrophicus* 1EJ7 possessed the obvious advantages for the highest reducing sugar content after pretreatment of switchgrass. Meanwhile, as there was no report on the cellulase from *B. methylotrophicus* 1EJ7, it was promising to found some novel cellulases and apply both the strain and cellulase to the degradation of the biomass in the industry.

It was noteworthy that different carbon substrates would induce different cellulases and further lead to different capabilities of reducing sugar production. When 8 strains were applied to hydrolyze different carbon source substrates, the strains exhibited a better performance in reducing sugar production in CMC-Na medium than that in Avicel, which was mainly attributed to two parts: 1. the different structure of cellulose substrates: Avicel was more difficult to hydrolyze than CMC-Na by cellulase for the unique microcrystalline structure [35]; 2. the different action modes of exocellulase and endocellulase: as the type of cellulolytic enzyme was critical to the hydrolysis of different cellulose substrates, the efficiency in hydrolyzing CMC or Avicel was also significantly affected by the enzyme types [36, 37].

Moreover, it was also found that the strains performing well in Avicel and CMC-Na degradation did not show a same good performance in hydrolyzing biomass, which was probably due to the complex network of lignin-hemicellulos-cellulose in biomass materials. It also needed to note that FPase and CMCase of different strains (Fig. 4b and c) exhibited similar cellulase activity in different mediums. Furthermore, except for *B. subtilis* 1AJ3 and the *B. subtilis* 3BJ4, Avicelase activity was detected in all mediums. Other strains could synthesize enzyme in limited carbon source medium pretreated by all other strains. An interesting phenomenon was also found that *B. subtilis* 1AJ2 could produce Avicelase enzyme only in CMC-Na medium, which indicated that the selected strains processed the function to synthesize enzyme in a limited carbon source medium. However, not all strains could be induced to produce Avicelase, which might be the reason why crystalline cellulose or more complex structure were hard to hydrolyze reported by the related study [38], on the other hand, the results further proved the special applicability of the selected stains.

Among the three biomass materials in present study, the pretreatment of switchgrass, considered as major non-food biomass resources originated from US [39], led to the highest production of reducing sugar. Compared with wheat straw and corn stover, switchgrass was a herbaceous plant with a less tight and crystalline structure and primarily composed of glucose than xylose or other monomeric sugars, which might be the main reason for the easily hydrolyzation by the strains, and a similar study also has been reported by Sharma R [40]. Compared with acid pretreatment or hot compress water pretreatment [41], bacteria cultivation was always considered as a gentle pretreatment method without any chemicals (such as salt ion, acid and alkali) or dramatic physical conditions (such as high temperature, high pressure and radiation). In this study, *B. methylotrophicus* 1EJ7 was used to pretreat switchgrass, and 0.95 mg/mL reducing sugar was finally obtained, which also needed to be improved before the application. Existing studies showed that the competitiveness of the strains was always improved by optimizing cultivation condition or cooperating with other enzymes, meanwhile, novel technologies were also feasible, including gene editing and cellulase cloning expression, which was preliminarily conducted as shown follows.

### Cellulase of *B. methylotrophicus*

When talking about *B. methylotrophicus,* the majority of studies mainly focused on the following parts: 1. Screening [42]; 2. Utilized as biosurfactant-producer and agricultural agent [43], bioflocculant [44], as well as biofertilizer or biocontrol agent [45]; 3. Cyclic lipopeptides [46] and lipopeptides [47]; 4. Production of enzymes, such as levansucrase [48], α-amylase [49], lactosylfructoside [50], and xylanase [51]. However, there were rare reports about cellulases from *B. methylotrophicus*, and just only two types of cellulase, 1,3-1,4-beta-glucanase [52] and carboxymethyl cellulase [33] had been reported, which were both obtained by the purification from strain cultivation broth. But so far, the heterogeneously cloning and expression of polypeptides or enzymes of *B. methylotrophicus* were not found.

Accordingly, in this study, the molecular biology methods were applied to clone and express cellulase from bacteria *B. methylotrophicus* 1EJ7. Fortunately, two cellulases were successfully cloned and expressed, and the cellulolytic enzyme activities of cloning peptide were also verified, which can be considered as the first study about the heterogeneously cloning and expression of the cellulases (β-glucosidase and endoglucanase) from *B. methylotrophicus* strain. Enzyme activities of recombined crude enzyme Bgl and Egl reached 1670.15 ± 18.94 U/mL and 0.130 ± 0.002 U/mL, respectively. According to the results of SDS-PAGE, the light color band of Egl can be speculated to exhibit a low expression level, which might lead to relative low enzyme activity (U/mL) of cellulase, however, the expression level was still higher than the CMCase in strain growth broth in this study or other *Bacillus* [53]. A high expression level of Bgl was also detected under the proposed expression conditions and a high crude enzyme activity was obtained. Based on the sequence alignment results, several enzymes of high similarity with Bgl were found. For examples, β-1,3–1,4-glucanase from *B. subtilis* 168 with a crude enzyme activity of 1366.48 U/mL had highest sequence similarity of 94% with Bgl [54], and a beta-glucosidase with a similar *Mw* (27.35 kDa) from *B. licheniformis* showed enzyme activity of 67.34 U/mL [55]. Importantly, all the reported similar Bgls did not possesses a better enzyme activity than the recombinant Bgl in the present study, even the industrial optimized beta-glucosidase (560.4 U/mL) [56] and other heterogeneous expressed β-1,4-glucosidase from bacteria (*Paenibacillus sp*. 5.8 U/mL [57]) and fungi (*Daldinia eschscholzii*, 3.21 U/mg [58]; *Trichoderma reesei*, 25.13 U/mL [59]). What’s more, the enzyme activity of the recombinant Bgl was tens of thousands of times than that from wild microorganism, such as *Penicillium pinophilum* KMJ601 (3.2 U/mL) [60] and *Bacillus sp.* AS3 (0.04 U/mL) [53]. It was an interesting scientific issue that what the reason for the differences in the enzyme activity of Bgl with a similarity amino acid sequence. It was deduced that although it was highly similarity in the amino acid sequence (for instance, 94% similarity of Bgl with β-glucanases from *B. subtilis* 168 in amino acid sequence), the different amino acids might be the important part for the enzyme protein structure or the active sites, such as the binding sites of enzyme with substrates, catalytic sites, etc. If the different amino acid located in the important part and significantly influenced the active sites structure of the protein, the activity of the enzyme also might be significantly influenced, although just several amino acids were different. Eventhough this was just a deduction about this phenomenon obtained by the comparation with other reported similar enzymes, it provided a valuable study direction to make sure the function of the different amino acid on the enzyme activity. In this view, directed mutagenesis or knocking down of the proposed sites in peptides can be conducted to study the influence of proposed amino acid on the enzyme structure and activity. Moreover, the relationship between the enzyme structure and the activity, and the mechanism of the catalytic process should be also deeply studied in the further study.

In addition, as the β-glucosidase and endoglucanase had been expressed successfully, the characterization and optimization of the proposed recombinant enzymes should be studied in the further study. Correspondingly, the present study provided new materials of both strain and the recombinant enzymes for the cellulose degradation related studies and promoted the bio-pretreatment of the cellulose applied to industry.

## Conclusions

Various of cellulose-degrading bacteria in rotten wood of Qinling Mountains were isolated and identified as well as the characterization of cellulose degradation. Among the isolated strains, *Bacillus* were the dominant species, in which *B. methylotrophicus* 1EJ7 processed the best ability for cellulose degradation. Meanwhile, the β-glucosidase and endoglucanase were successfully heterogeneously cloned and expressed from *B. methylotrophicus* for the first time, which were also verified having well activity for cellulose degradation. In addition, the characterization, enzyme activity improvement and the mechanism of the cellulose degradation about the recombinant cellulases would be further studied.

## Materials and methods

### Materials

Five rotten wood samples (weed tree, red birch, poplar, alpine rhododendron and willow) were collected from Qinling Mountain in Shaanxi Province, China. Samples were transported to the laboratory and stored at 4 °C. Wheat straw, corn stover, switchgrass was washed by water and dried at 60 °C, and then crushed by high-peed pulverizer to 40 mesh. The chemicals used in this study were purchased from Kelong Chemical Reagent Chengdu Co., Aladdin or Sigma.

### Cellulolytic bacteria isolation and identification

Each sample was broken into pieces, and took 1 g added into LB medium (10 g/L NaCl, 10 g/L tryptone and 5 g/L yeast extract), then incubated at 37 °C for 24 h with a constant shaking speed of 120 rpm. The bacteria suspension was respectively transferred to two selective media. The two selective media, CM and AM, were used CMC-Na and Avicel as single carbon source separately, contained of 2.0 g/L sodium carboxymethyl cellulose (CMC-Na) or Avicel (PH-101), and others of 2.0 g/L (NH_4_)_2_SO_4_, 0.5 g/L MgSO_4_•7H_2_O, 1.0 g/L K_2_HPO_4_ at natural pH of 7.20 were the same. The strains were cultured for 48 h at 37 °C with 120 rpm before being spread on the selective media agar plates with 0.4 g/L Congo red. Plates were incubated at 37 °C for 72 h, and then different colonies on the plates were picked.

The strains which probably could produce cellulolytic enzymes had a hydrolyzed circle around the colony. 10 μL broth of each isolated strains was dripped on the Congo red agar plates and the hydrolysis circle diameters of were measured to primarily evaluate the cellulolytic capability. The selected strains were shown in Additional file 1: Figure S1.

The strains were cultured in broth for 48 h, then the cells were harvested and subjected to genome DNA extraction by a DNA extraction kit (Sangon Biotech, Shanghai, China). The universal primers of 27F and 1492R were utilized to amplify the 16S rRNA gene fragments. Polymerase chain reaction (PCR) was performed in a 25 μL reaction system containing 1 μL DNA template, 1 μL upstream primer (10 μM), 1 μL downstream primer (10 μM), 12 μL mixture, and 10 μL double-distilled water. The PCR amplification was performed as follows: initial denaturation at 95 °C for 5 min; 35 cycles of 94 °C for 1 min, 58 °C for 30 s, and 72 °C for 1 min; and final extension at 72 °C for 10 min.

Agarose gel electrophoresis was used to confirm target products and the PCR products were sequenced. The sequences were applied to BLAST on the NCBI database (http://blast.ncbi.nlm.nih.gov/). The 16S rRNA gene sequences have been submitted to GenBank (Accession Numbers showed in Additional file 9). Phylogenetic tree was constructed in iTOL (http://itol.embl.de).

### Reducing sugar determination and enzymatic activity assay

Fifty mL of the two selective media were individually transferred into 100 mL flasks and autoclaved under 121 °C for 20 min. The flasks were inoculated with 6% (v/v) seed bacteria (dilute broth OD_600_ to 1.0) and grow at 37 °C for 48 h under 120 rpm. The cell-free supernatant was obtained by centrifugation (11,000 rpm, 4 °C, and 10 min) to examine reducing sugar content and the activities of crude cellulase. The FPase (filter paper activity), CMCase activity, and Avicelase activity were analyzed according to the methods described [61]. All cellulase activities were determined at 50 °C. Reducing sugar of each cultivated liquid was analyzed by DNS method [62]. One unit (U) of FPase/CMCase/Avicelase were defined as the amount of enzyme that produce 1 μmol of glucose per minute under standard conditions. The activities of recombinant β-glucosidase was determined by p-NPG (p-4-nitrophenyl β-D-glucopyranoside) as substrate at 50 °C, 10 min with p-NP as standard [63]. One unit (U) of β-glucosidase enzyme was defined as the amount of enzyme required to release 1 μmol of p-NP per minute.

### Cultivation in different carbon sources

Different carbon source of wheat straw (4.0 g/L), corn stover (4.0 g/L), switchgrass (4.0 g/L), Avicel (2.0 g /L) or CMC-Na (2.0 g/L) was used as sole carbon source. Reducing sugar and cellulase activity were measured.

### Single and mixed strain cultivation of cellulosic materials without pretreatment

Three bacteria strains (*B. methylotrophicus* 1EJ7, *B. subtilis* 1AJ3 and *B. subtilis* 3BJ4) were selected to degrade wheat straw, corn stover or switchgrass by single and mixture owing to their higher cellulolytic activity. Mixture of the three strains (1:1:1) had the same inoculum size as the single strain. The inoculum size of the single strain or mixed strains was 6%. The concentrations of wheat straw, corn stover, and switchgrass were 7% (w/v), after which they were grown at 37 °C, 120 rpm for 72 h. Reducing sugar was determined at intervals of 12 h.

### Scanning electron microscopy (SEM) and X-ray diffraction

*B. methylotrophicus* 1EJ7 was utilized to hydrolyze wheat straw without pretreatment as an example to show morphology changes before and after hydrolysis by SEM method [64].

X-ray diffraction (Xian Asn Tech) was used to show wheat straw physical structures with diffraction angles spanned from 2*θ* = 5–50°. The radiation was generated at a voltage of 40 kV with a current of 35 mA, and by a scan step size of 0.033° [22]. Crystallinity Index Cr*I* (%) = [(*I*_002_ - *I*_am_)/*I*_002_] × 100 (*I*_002_ is the intensity of crystalline portion of cellulose at 2*θ* = 22°, and *I*_am_ is the peak intensity of the amorphous portion at 2θ = 18°).

### Cloning and expression of cellulase gene from *B. methylotrophicus* 1EJ7 in *E. coli*

*B. methylotrophicus* 1EJ7 was cultured in LB medium at 37 °C for 24 h under 150 rpm. The cells were collected by centrifugation (10,000 rpm, 4 °C, and 10 min) and the genomic DNA was extracted using an Ezup column bacteria genomic DNA purification kit (Shanghai Sangon Biotech Co., Ltd.). The extracted DNA was used as a template for PCR amplification. The genes encoding β-glucosidase and endoglucanase were amplified by using primers based on the gene sequences of the β-glucosidase of *Bacillus velezensis* AS43.3 (CP003838.1) and endoglucanase of *Bacillus velezensis* strain JTYP2 (CP020375.1). The gene encoding the β-glucosidase was amplified by PCR (94 °C for 5 min, and then 35 cycles of 94 °C for 1 min, 65 °C for 1 min (− 0.5 °C/c), 72 °C 3 min, and 72 °C for 10 min) with a forward primer of 5′- CATG*CCATGG*GCATGTTTTATCGTATGAAACGAGTG (*Nco*I site was underlined) and a reverse primer 5′-CCG*CTCGAG*TTTTTTTGTATAGCGCACCCA (*Xho*I site was underlined) using a Takara ExTaqHS (Takara Bio, Shiga, Japan). The gene encoding the endoglucanase was amplified under the same PCR condition described above with a forward primer of 5′-CATG*CCATGG*GCATGAAACGGTCAATTTCTATTTTT (*Nco*I site was underlined) and a reverse primer of 5′-CCG*CTCGAG*ATTGGGTTCTGTTCCCCAAA (*Xho*I site was underlined). The amplified genes were double digested with *Nco*I and *Xho*I, and inserted into the corresponding site of the pET-28a(+) vector (Novagen) by T4 ligase.

Then, the constructed plasmid was transformed into *E.coli* BL21 (DE3) by hot hit under 42 °C for 90s and correct transformants were identified by PCR amplification and sequencing. The transformant was cultured in 1 L LB medium containing 1 mg/mL kanamycin at 37 °C until the absorbance at 600 nm reached 0.6. After that, expression was induced by adding a final density of 0.2 mM IPTG, and the transformant was further cultured at 25 °C for 16 h. The cells were collected by centrifuging (8000×*g*, 4 °C, 10 min), and then suspended in PBS buffer (pH 7.2). Cells were disrupted by ultrasonication under 300 W output power, a repeating cycle of 1 s ultrasonic treatment and 5 s shutdown, for 60 min on a SCIENTZ-IID ultrasonic homogenizer (Ningbo Scientz Biotechnology Polytron Technologies Inc. Zhejiang province, China). The resulting cell lysates were centrifuged (8000×*g*, 4 °C, 30 min). A 15% SDS-PAGE was performed to analyze the supernatant and the insoluble fraction of the sonicated whole cell lysate.

### Bioinformatic analysis and homology modeling

The plasmids of pET-28a-Bgl and pET-28a-Egl were sequenced. The primary sequences of Bgl and Egl protein were obtained by amino acid translation software, and the homology templates were obtained through retrieving in the protein database PDB. Physiochemical characteristics were predicted on ExPASy (http://web.expasy.org/protparam/). Conserved domain was analyzed by CDD of NCBI (https://www.ncbi.nlm.nih.gov/cdd). Clustal Omega (https://www.ebi.ac.uk/Tools/msa/clustalo/) was used for sequence alignments. Secondary structure and 3D structure were predicted by PSIPRED (http://bioinf.cs.ucl.ac.uk/psipred/) and I-TASSER (https://zhanglab.ccmb.med.umich.edu/I-TASSER/).

## Supplementary information

**Additional file 1: Figure S1.** Hydrolyzed circle of isolates on the Congo red agar plate. (a) Plates with CMC-Na as the sole carbon source. (b) Plates with Avicel as the sole carbon source

**Additional file 2: Table S2.** Isolated strains growth situation and clear zone size on Congo red plates

**Additional file 3: Figure S3.** Reducing sugar production and enzyme activities in CMC-Na (a) and Avicel (b) as sole carbon source medium

**Additional file 4: Table S4**-**1.** p-NP standard curve. **Table S4**-**2.** Bgl enzyme activity. **Table S4**-**3.** Glucose standard curve. **Table S4-4.** Egl enzyme activity.

**Additional file 5: Figure S5.** Domain analysis of Bgl

**Additional file 6: Figure S6.** Domain analysis of Egl

**Additional file 7: Figure S7.** Amino acid sequence alignments for Bgl and comparison with same gene in different strains. In which Bgl stand for β-glucosidase in this study; AS43.3 for β-glucosidase gene of *Bacillus velezensis* AS43.3 (CP003838.1) (complete gene); JTYP2 for *Bacillus velezensis* JTYP2 (CP020375.1), and 168 for *Bacillus subtlis* 168 (AL009126.3)

**Additional file 8: Figure S8.** Amino acid sequence alignments for Egl and comparison with same gene in different strains. In which Egl stand for endoglucanase in this study; JTYP2 for endoglucanase gene of *Bacillus velezensis* JTYP2 (CP020375.1) (complete gene); AS43.3 for *Bacillus velezensis* AS43.3 (CP003838.1), and 168 for *Bacillus subtlis* 168 (AL009126.3)

**Additional file 9.** Accession Numbers

## Data Availability

All data generated or analyzed during this study are included in this published article and its supplementary information files.

## Abbreviations

CMC-Na: Sodium carboxymethyl cellulose
FPase: Filter paper activity
NCBI: National center for biotechnology information
PCR: Polymerase chain reaction
p-NPG: p-4-nitrophenyl β-D-glucopyranoside
SEM: Scanning electron microscopy
