# Supplementary material for: Screening of cellulolytic bacteria from rotten wood of Qinling (China) for biomass degradation and cloning of cellulases from Bacillus methylotrophicus
Source: BMC Biotechnol. 2020 Jan 7;20:2. doi: 10.1186/s12896-019-0593-8 (PMC6947901; doi:10.1186/s12896-019-0593-8)
Supplement: Supplementary file 1 — Additional file 1: Figure S1. Hydrolyzed circle of isolates on the Congo red agar plate. (a) Plates with CMC-Na as the sole carbon source. (b) Plates with Avicel as the sole carbon source [file 12896_2019_593_MOESM1_ESM.docx]

**Supplementary 1**

**
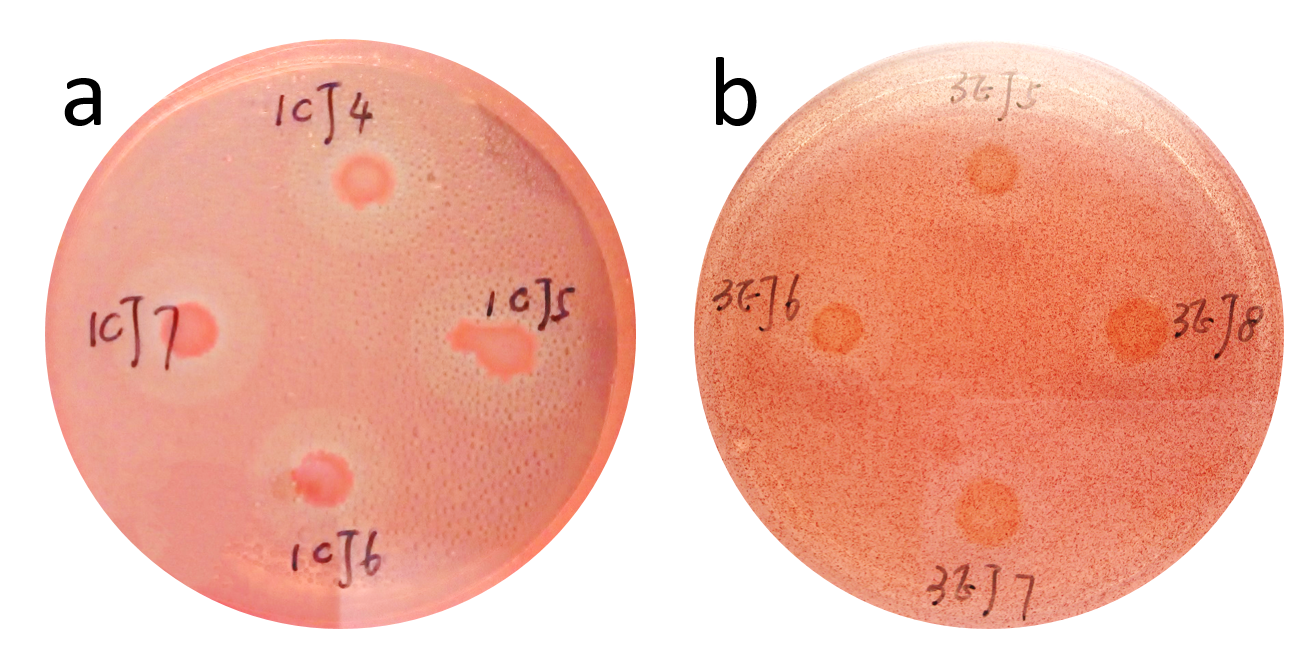
**

**Fig. S1** Hydrolyzed circle of isolates on the Congo red agar plate. (a) Plates with CMC-Na as the sole carbon source. (b) Plates with Avicel as the sole carbon source.
