# Supplementary material for: Screening of cellulolytic bacteria from rotten wood of Qinling (China) for biomass degradation and cloning of cellulases from Bacillus methylotrophicus
Source: BMC Biotechnol. 2020 Jan 7;20:2. doi: 10.1186/s12896-019-0593-8 (PMC6947901; doi:10.1186/s12896-019-0593-8)
Supplement: Supplementary file 2 — Additional file 2: Table S2. Isolated strains growth situation and clear zone size on Congo red plates [file 12896_2019_593_MOESM2_ESM.docx]

**Supplementary 1**

**
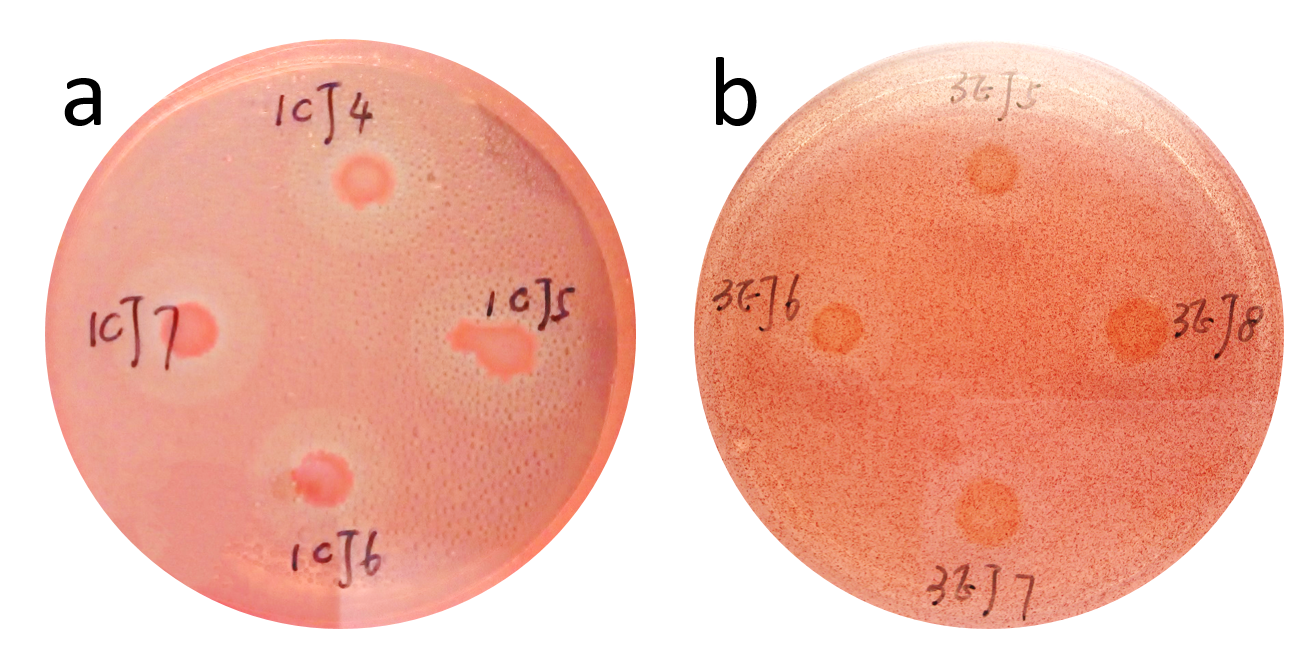
**

**Fig. S1** Hydrolyzed circle of isolates on the Congo red agar plate. (a) Plates with CMC-Na as the solo carbon source. (b) Plates with Avicel as the solo carbon source.

**Supplementary 2**

**Table S2** Isolated strains growth situation and clear zone size on Congo red plates

| Source organism | No. | Solo carbon source | Diameter of strain colony (mm) | Diameter of clear zone (mm) | Ratio of clear zone and strain diameter |
| --- | --- | --- | --- | --- | --- |
| Weed tree | 1AJ1 | CMC-Na | 8.0 | 20 | 2.50 |
|  | 1AJ2 | CMC-Na | 7.5 | 20 | 2.67 |
|  | 1AJ3 | CMC-Na | 7.5 | 19 | 2.53 |
|  | 1AJ4 | CMC-Na | 8.0 | 21 | 2.63 |
|  | 3AJ1 | Avicel | 7.0 | 22 | 3.14 |
|  | 3AJ5 | Avicel | 6.0 | 19 | 3.17 |
|  | 3AJ4 | Avicel | 7.0 | 15 | 2.14 |
|  | 3AJ7 | Avicel | 6.0 | 19 | 3.17 |
| Red birch | 1BJ1 | CMC-Na | 7.0 | 21 | 3.00 |
|  | 1BJ2 | CMC-Na | 8.0 | 21 | 2.63 |
|  | 1BJ3 | CMC-Na | 8.5 | 21 | 2.47 |
|  | 1BJ4 | CMC-Na | 9.0 | 20 | 2.22 |
|  | 1BJ5 | CMC-Na | 9.0 | 21.5 | 2.39 |
|  | 1BJ6 | CMC-Na | 8.5 | 22 | 2.59 |
|  | 1BJ7 | CMC-Na | 9.0 | 22 | 2.44 |
|  | 1BJ8 | CMC-Na | 7.5 | 20 | 2.67 |
|  | 1BJ9 | CMC-Na | 7.0 | 21 | 3.00 |
|  | 3BJ2 | Avicel | 6.0 | 17 | 2.83 |
|  | 3BJ3 | Avicel | 5.5 | 16.5 | 3.00 |
|  | 3BJ4 | Avicel | 8.0 | 19 | 2.38 |
|  | 3BJ5 | Avicel | 6.5 | 19 | 2.92 |
|  | 3BJ6 | Avicel | 7.5 | 16 | 2.13 |
|  | 3BJ7 | Avicel | 6.5 | 16 | 2.46 |
|  | 3BJ8 | Avicel | 6.0 | 12 | 2.00 |
|  | 3BJ9 | Avicel | 6.0 | 11 | 1.83 |
| Poplar | 1CJ1 | CMC-Na | 8.5 | 25 | 2.94 |
|  | 1CJ2 | CMC-Na | 8.0 | 21 | 2.63 |
|  | 1CJ3 | CMC-Na | 9.0 | 25 | 2.78 |
|  | 1CJ4 | CMC-Na | 8.5 | 20 | 2.35 |
|  | 1CJ5 | CMC-Na | 9.0 | 24 | 2.67 |
|  | 1CJ6 | CMC-Na | 8.5 | 18.5 | 2.18 |
|  | 1CJ7 | CMC-Na | 8.0 | 19 | 2.38 |
|  | 1CY1 | CMC-Na | 8.0 | 11 | 1.38 |
|  | 1CY2 | CMC-Na | 9.0 | 11.5 | 1.28 |
|  | 1CY3 | CMC-Na | 9.0 | 11.5 | 1.28 |
|  | 3CJ5 | Avicel | 4.0 | - | - |
|  | 3CJ6 | Avicel | 9.0 | 18 | 2.00 |
|  | 3CJ7 | Avicel | 5.5 | 12 | 2.18 |
|  | 3CJ8 | Avicel | 6.5 | 18 | 2.77 |
|  | 3CJ9 | Avicel | 7.5 | 16 | 2.13 |
|  | 3CJ10 | Avicel | 6.0 | 9 | 1.50 |
|  | 3CY2 | Avicel | 5.0 | 9 | 1.80 |
|  | 3CY3 | Avicel | 7.0 | 9 | 1.29 |
|  | 3CY6 | Avicel | 6.5 | 8 | 1.23 |
| Alpine rhododendron | 1DJ1 | CMC-Na | 8.0 | 11 | 1.38 |
|  | 1DJ2 | CMC-Na | 9.0 | 11 | 1.22 |
|  | 1DJ3 | CMC-Na | 8.0 | 11 | 1.38 |
|  | 1DJ4 | CMC-Na | 8.0 | 11 | 1.38 |
|  | 1DJ5 | CMC-Na | 8.0 | 10.5 | 1.31 |
|  | 1DJ6 | CMC-Na | 7.5 | 14 | 1.87 |
|  | 3DJ1 | Avicel | 6.5 | 13 | 2.00 |
|  | 3DJ2 | Avicel | 6.0 | 12 | 2.00 |
|  | 3DJ4 | Avicel | 6.0 | 11 | 1.83 |
|  | 3DJ5 | Avicel | 6.5 | 11 | 1.69 |
|  | 3DJ6 | Avicel | 7.0 | 13 | 1.86 |
|  | 3DJ7 | Avicel | 6.0 | 13 | 2.17 |
|  | 3DY1 | Avicel | 8.0 | 9 | 1.13 |
|  | 3DY2 | Avicel | 7.0 | 10 | 1.43 |
|  | 3DY3 | Avicel | 7.0 | 11 | 1.57 |
| Willow | 1EJ1 | CMC-Na | 9.0 | 16 | 1.78 |
|  | 1EJ2 | CMC-Na | 7.5 | 11.5 | 1.53 |
|  | 1EJ3 | CMC-Na | 7.5 | 14 | 1.87 |
|  | 1EJ4 | CMC-Na | 7.5 | 13 | 1.73 |
|  | 1EJ5 | CMC-Na | 8.0 | 15 | 1.88 |
|  | 1EJ6 | CMC-Na | 9.0 | 12 | 1.33 |
|  | 1EJ7 | CMC-Na | 7.0 | 19 | 2.71 |
|  | 1EY1 | CMC-Na | 8.0 | 11 | 1.38 |
|  | 1EY2 | CMC-Na | 6.5 | 9 | 1.38 |
|  | 1EY8 | CMC-Na | 8.0 | 9 | 1.13 |
|  | 3EJ1 | Avicel | 7.5 | 14 | 1.87 |
|  | 3EJ2 | Avicel | 9.0 | 19 | 2.11 |
|  | 3EJ3 | Avicel | 8.0 | 18 | 2.25 |
|  | 3EJ4 | Avicel | 8.5 | 18 | 2.12 |
|  | 3EJ5 | Avicel | 8.0 | 11 | 1.38 |
|  | 3EJ6 | Avicel | 8.0 | 7 | 0.88 |
|  | 3EJ7 | Avicel | 9.0 | 11 | 1.22 |
|  | 3EJ8 | Avicel | 9.0 | 14 | 1.56 |
|  | 3EY1 | Avicel | 8.0 | 11 | 1.38 |
|  | 3EY2 | Avicel | Point colony | 7 | - |
|  | 3EY3 | Avicel | 8.0 | 11 | 1.38 |
|  | 3EY4 | Avicel | 8.5 | 14 | 1.65 |

**S****upplementary 3**


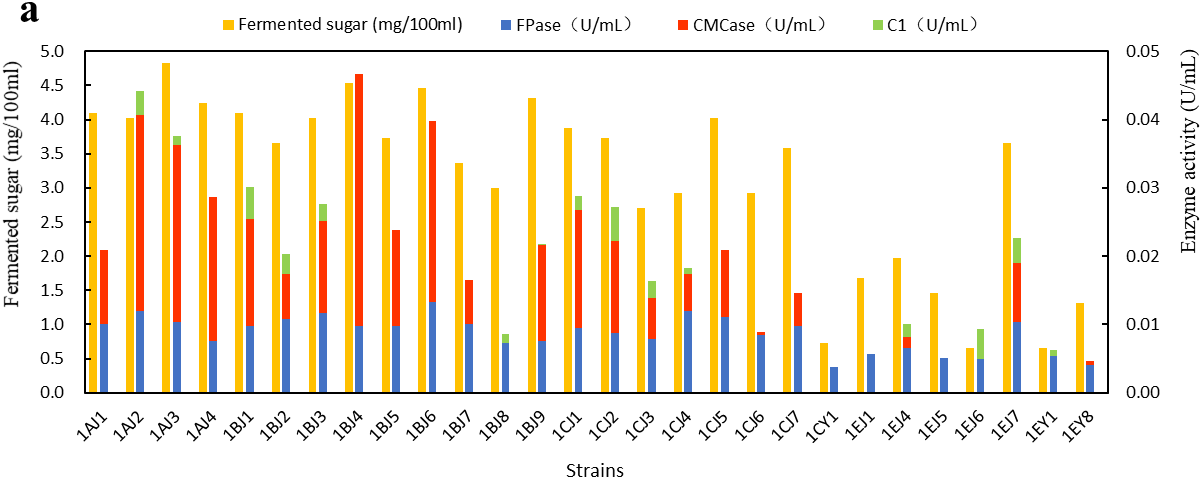

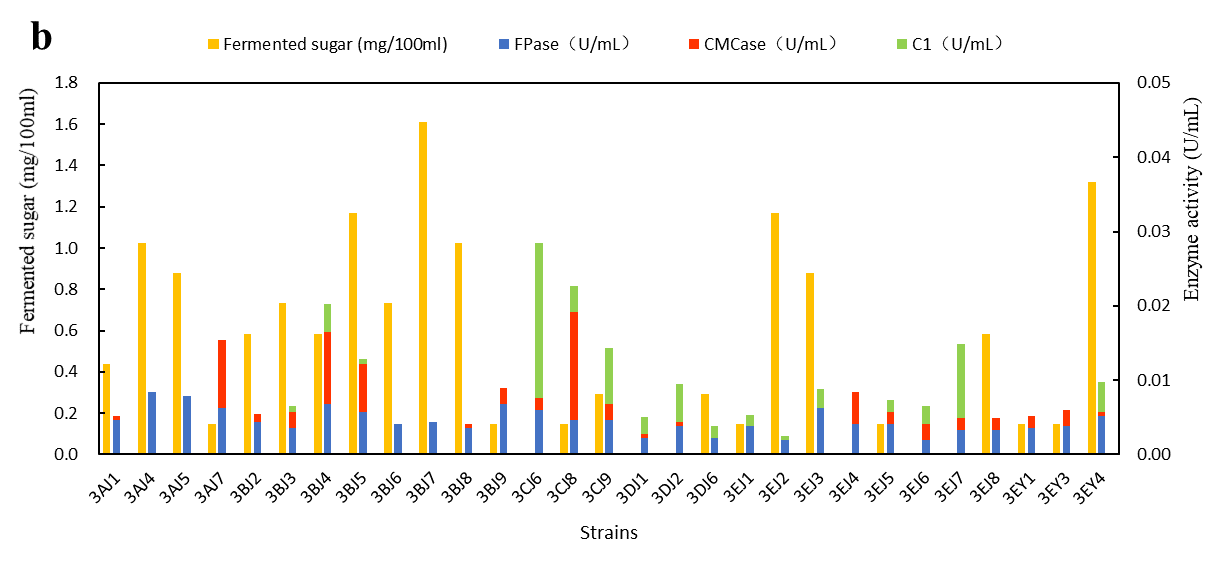


Fig.S3 Reducing sugar production and enzyme activities in CMC-Na (a) and Avicel (b) as sole carbon source medium.

**Supplementary 4**

**Recombined enzyme activity**

**Bgl**

**Table S4-1 p-NP standard curve**

| p-NP(mM) | 0.025 | 0.050 | 0.075 | 0.100 | 0.125 | 0.150 | 0.175 | 0.200 | 0.225 |
| --- | --- | --- | --- | --- | --- | --- | --- | --- | --- |
| OD410 | 0.190 | 0.336 | 0.463 | 0.604 | 0.731 | 0.878 | 1.003 | 1.117 | 1.259 |

**Y=5.3153X+0.0668 R^2^=0.999**

**Measured method:**

5μL crude enzyme + 20μL 25mM p-NPG + 115μL PBS →50℃, 10min→70μL 0.4M Na2CO3→OD410

**Table S4-2 Bgl enzyme activity**

| Blank | OD410 | U/mL | U_average_/mL | SD |
| --- | --- | --- | --- | --- |
| 0.139 | 0.645 | 1652.588 | 1670.147 | 18.93862 |
| 0.142 | 0.652 | 1667.639 |  |  |
| 0.142 | 0.658 | 1690.215 |  |  |

**Egl**

**Table S4-3 Glucose standard curve**

| Glucose content(mg) | 0.000 | 0.010 | 0.020 | 0.030 | 0.040 | 0.050 | 0.060 | 0.070 | 0.080 |
| --- | --- | --- | --- | --- | --- | --- | --- | --- | --- |
| OD540 | 0.109 | 0.131 | 0.316 | 0.519 | 0.725 | 0.906 | 1.142 | 1.323 | 1.544 |

**Y=20.21X-0.0837 R^2^=0.999**

**Measured method:**

50μL crude enzyme + 50μL PBS +100μL 1%CMC-Na substance→50℃,30min→300μLDNS→100℃,5min→OD540

**Table S4-4 Egl enzyme activity**

| Blank | OD540 | U/mL | Uaverage/mL | SD |
| --- | --- | --- | --- | --- |
| 0.112 | 0.754 | 0.132845 | 0.130343 | 0.002318 |
| 0.115 | 0.732 | 0.128268 |  |  |
| 0.113 | 0.739 | 0.129916 |  |  |

**Supplementary 5**
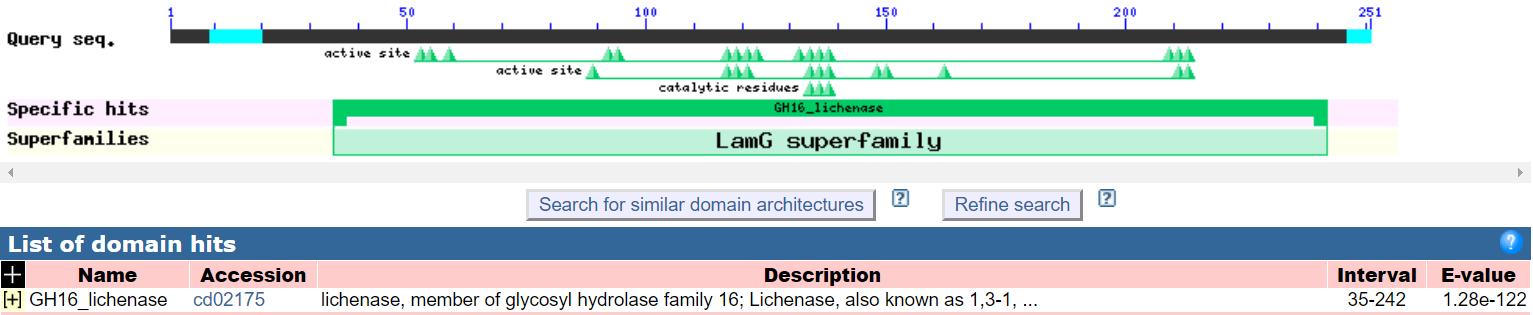


Figure S5 Domain analysis of Bgl


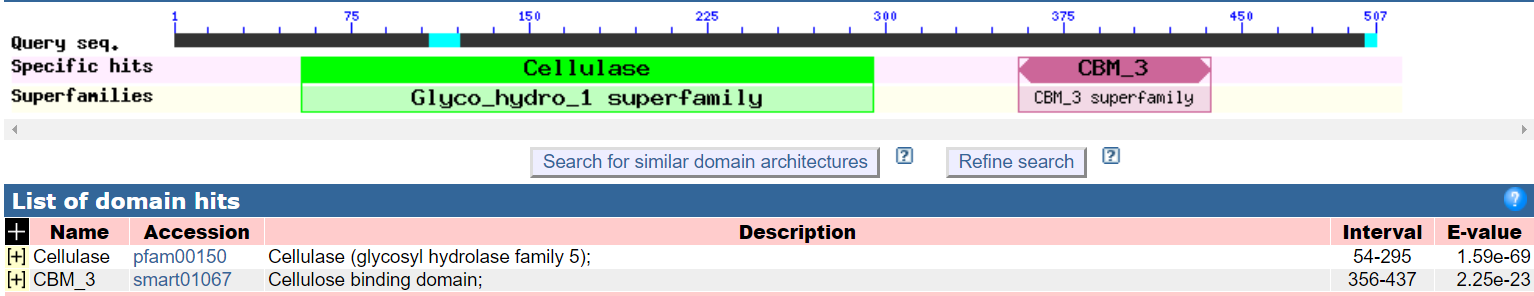
**Supplementary 6**

Figure S6 Domain analysis of Egl

**Supplementary 7**

**
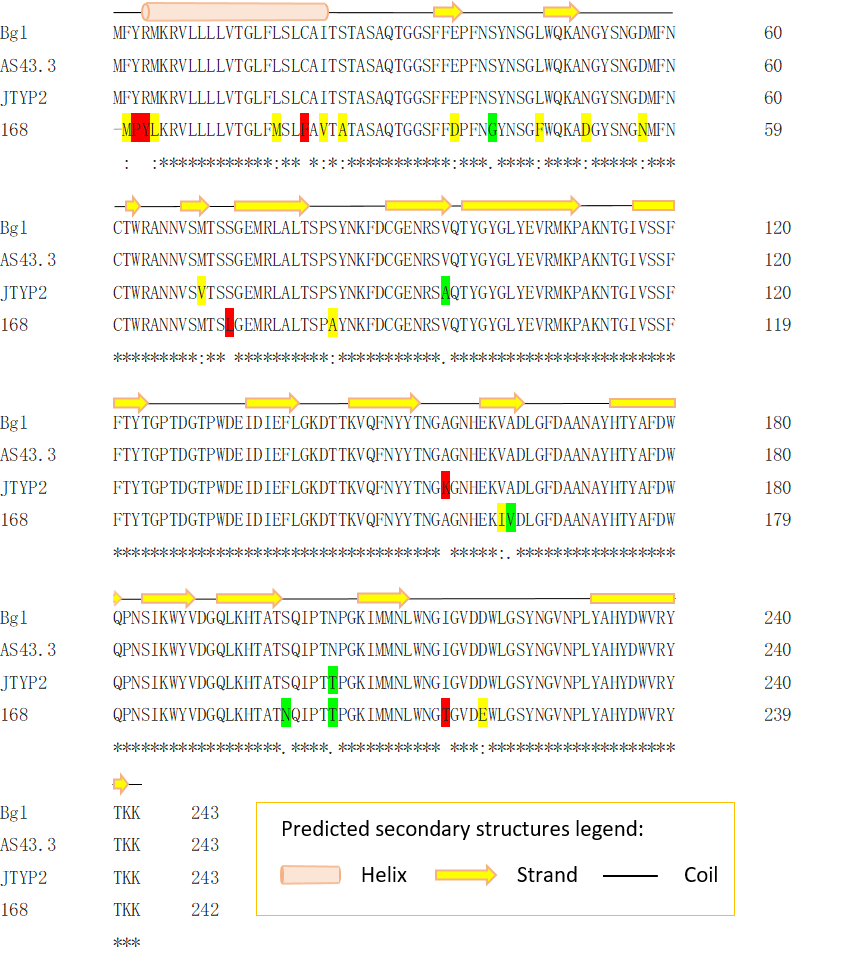
**

Figure S7 Amino acid sequence alignments for Bgl and comparison with same gene in different strains. In which Bgl stand for β-glucosidase in this study; AS43.3 for β-glucosidase gene of *Bacillus velezensis* AS43.3 (CP003838.1) (complete gene); JTYP2 for *Bacillus velezensis* JTYP2 (CP020375.1), and 168 for *Bacillus subtlis* 168 (AL009126.3).

Protein secondary structure of Bgl was predicted by PSIPRED. Helix, strand and coil was showed on top of aa sequence.

**
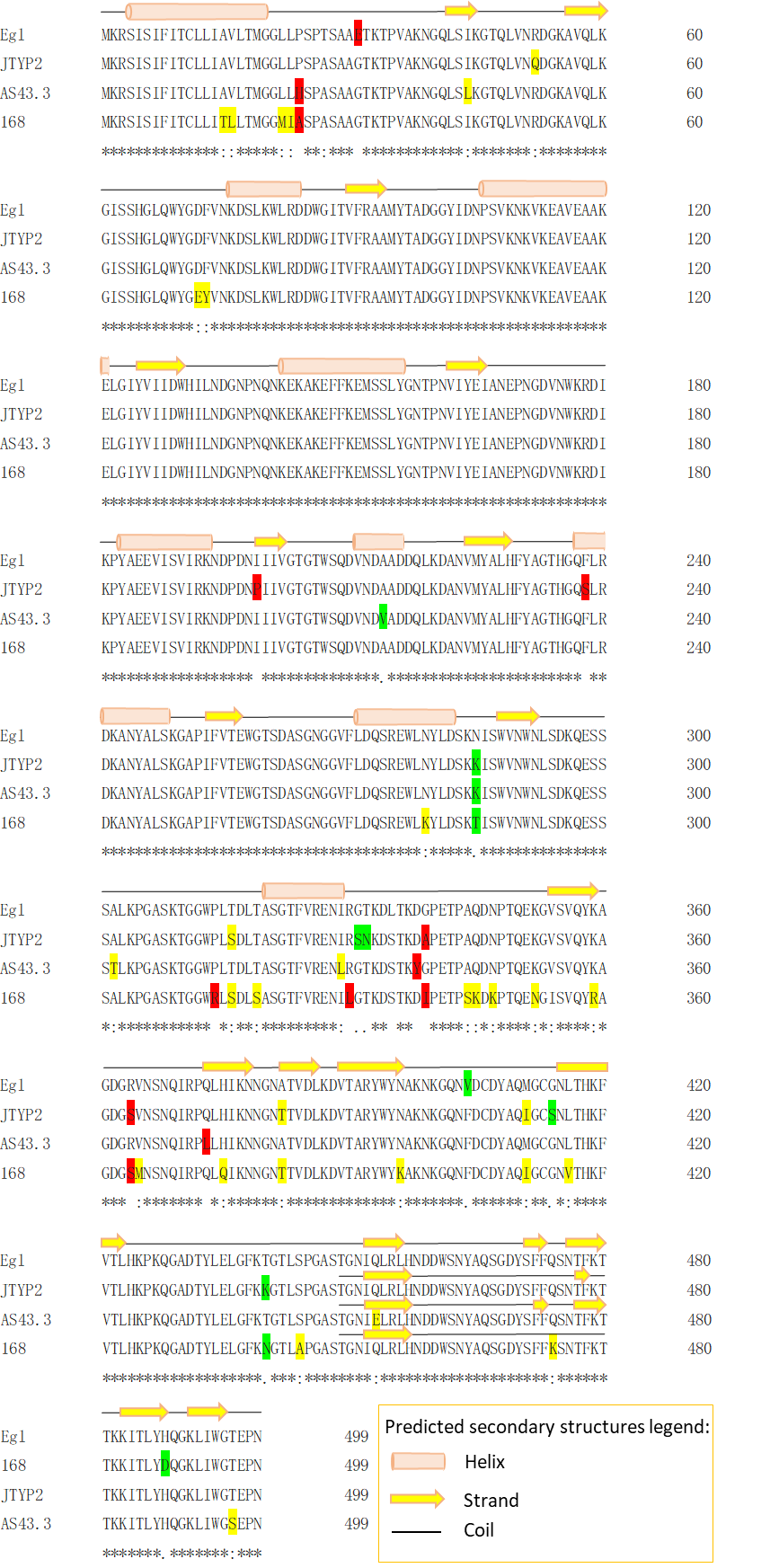
Supplementary 8**

Figure S8 Amino acid sequence alignments for Egl and comparison with same gene in different strains. In which Egl stand for endoglucanase in this study; JTYP2 for endoglucanase gene of *Bacillus velezensis* JTYP2 (CP020375.1) (complete gene); AS43.3 for *Bacillus velezensis* AS43.3 (CP003838.1), and 168 for *Bacillus subtlis* 168 (AL009126.3).

Protein secondary structure of Egl was predicted by PSIPRED. Helix, strand and coil was showed on top of aa sequence.

**Supplementary 9**

**Accession Numbers**

strain 1AJ1: MG062799, strain 1AJ2: MG062800, strain 1AJ3: MG062801, strain 1AJ4: MG062802, strain 1BJ1: MG062803, strain 1BJ2: MG062804, strain 1BJ3: MG062805, strain 1BJ4: MG062806, strain 1BJ6: MG062808, strain 1BJ7: MG062809, strain 1BJ8: MG062810, strain 1BJ9: MG062811, strain 1CJ1: MG062812, strain 1CJ2: MG062813, strain 1CJ3: MG062814, strain 1CJ4: MG062815, strain 1CJ5: MG062816, strain 1CJ6: MG062817, strain 1CJ7: MG062818, strain 1CY1: MG062819, strain 1EJ1: MG062820, strain 1EJ4: MG062821, strain 1EJ6: MG062823, strain 1EJ7: MG062824, strain 1EY1: MG062825, strain 1EY8: MG062826, strain 3AJ1: MG062827, strain 3AJ4: MG062828, strain 3AJ5: MG062829, strain 3AJ7: MG062830, strain 3BJ2: MG062831, strain 3BJ3: MG062832, strain 3BJ4: MG062833, strain 3BJ5: MG062834, strain 3BJ6: MG062835, strain 3BJ7: MG062836, strain 3BJ8: MG062837, strain 3BJ9: MG062838, strain 3CJ6: MG062839, strain 3CJ8: MG062840, strain 3CJ9: MG062841, strain 3DJ1: MG062842, strain 3DJ2: MG062843, strain 3DJ6: MG062844, strain 3EJ1: MG062845, strain 3EJ2: MG062846, strain 3EJ3: MG062847, strain 3EJ4: MG062848, strain 3EJ5: MG062849, strain 3EJ6: MG062850, strain 3EJ7: MG062851, strain 3EJ8: MG062852, strain 3EY1: MG062853, strain 3EY3: MG062854, strain 3EY4: MG062855
