# Supplementary material for: Screening of cellulolytic bacteria from rotten wood of Qinling (China) for biomass degradation and cloning of cellulases from Bacillus methylotrophicus
Source: BMC Biotechnol. 2020 Jan 7;20:2. doi: 10.1186/s12896-019-0593-8 (PMC6947901; doi:10.1186/s12896-019-0593-8)
Supplement: Supplementary file 3 — Additional file 3: Figure S3. Reducing sugar production and enzyme activities in CMC-Na (a) and Avicel (b) as sole carbon source medium [file 12896_2019_593_MOESM3_ESM.docx]

**S****upplementary 3**


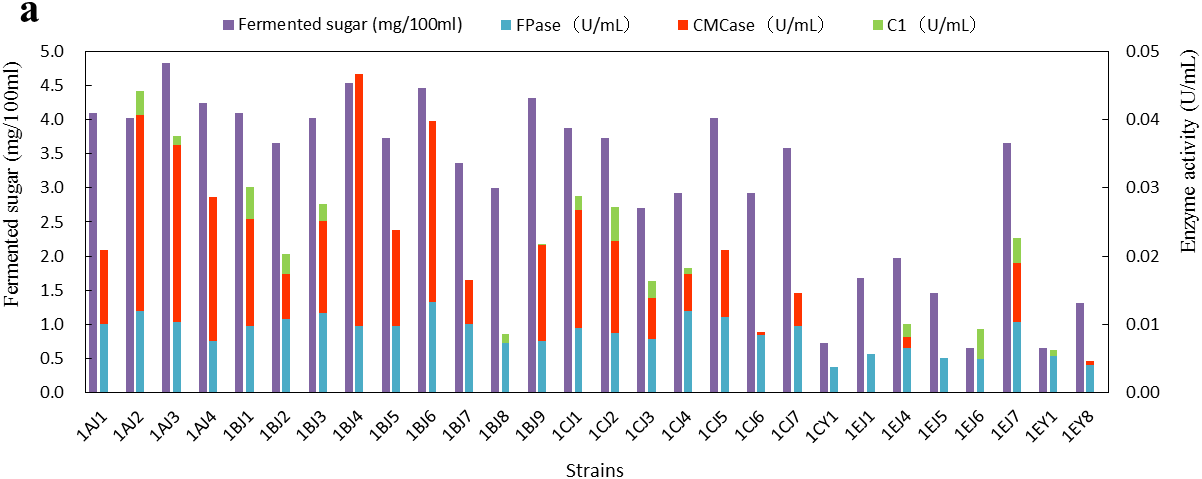

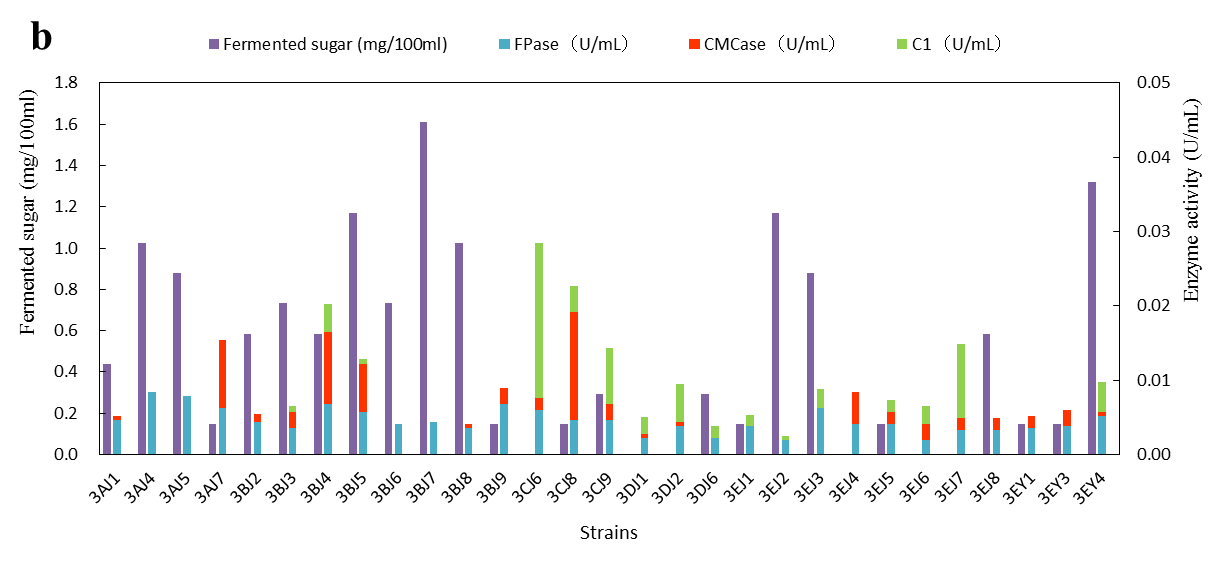


Fig.S3 Reducing sugar production and enzyme activities in CMC-Na (a) and Avicel (b) as sole carbon source medium.
