# Supplementary material for: Screening of cellulolytic bacteria from rotten wood of Qinling (China) for biomass degradation and cloning of cellulases from Bacillus methylotrophicus
Source: BMC Biotechnol. 2020 Jan 7;20:2. doi: 10.1186/s12896-019-0593-8 (PMC6947901; doi:10.1186/s12896-019-0593-8)
Supplement: Supplementary file 4 — Additional file 4: Table S4-1. p-NP standard curve. Table S4-2. Bgl enzyme activity. Table S4-3. Glucose standard curve. Table S4-4. Egl enzyme activity. [file 12896_2019_593_MOESM4_ESM.docx]

**Supplementary 4**

**Recombined enzyme activity**

**Bgl**

Table S4-1 p-NP standard curve

| p-NP(mM) | 0.025 | 0.050 | 0.075 | 0.100 | 0.125 | 0.150 | 0.175 | 0.200 | 0.225 |
| --- | --- | --- | --- | --- | --- | --- | --- | --- | --- |
| OD410 | 0.190 | 0.336 | 0.463 | 0.604 | 0.731 | 0.878 | 1.003 | 1.117 | 1.259 |

**Y=5.3153X+0.0668 R^2^=0.999**

**Measured method:**

5μL crude enzyme + 20μL 25mM p-NPG + 115μL PBS →50℃, 10min→70μL 0.4M Na2CO3→OD410

Table S4-2 Bgl enzyme activity

| Blank | OD410 | U/mL | U_average_/mL | SD |
| --- | --- | --- | --- | --- |
| 0.139 | 0.645 | 1652.588 | 1670.147 | 18.93862 |
| 0.142 | 0.652 | 1667.639 |  |  |
| 0.142 | 0.658 | 1690.215 |  |  |

**Egl**

Table S4-3 Glucose standard curve

| Glucose content(mg) | 0.000 | 0.010 | 0.020 | 0.030 | 0.040 | 0.050 | 0.060 | 0.070 | 0.080 |
| --- | --- | --- | --- | --- | --- | --- | --- | --- | --- |
| OD540 | 0.109 | 0.131 | 0.316 | 0.519 | 0.725 | 0.906 | 1.142 | 1.323 | 1.544 |

**Y=20.21X-0.0837 R^2^=0.999**

**Measured method:**

50μL crude enzyme + 50μL PBS +100μL 1%CMC-Na substance→50℃,30min→300μLDNS→100℃,5min→OD540

Table S4-4 Egl enzyme activity

| Blank | OD540 | U/mL | Uaverage/mL | SD |
| --- | --- | --- | --- | --- |
| 0.112 | 0.754 | 0.132845 | 0.130343 | 0.002318 |
| 0.115 | 0.732 | 0.128268 |  |  |
| 0.113 | 0.739 | 0.129916 |  |  |
