# Supplementary figures and images for: Screening of cellulolytic bacteria from rotten wood of Qinling (China) for biomass degradation and cloning of cellulases from Bacillus methylotrophicus
Source: BMC Biotechnol. 2020 Jan 7;20:2. doi: 10.1186/s12896-019-0593-8 (PMC6947901; doi:10.1186/s12896-019-0593-8)

**Supplementary 5**
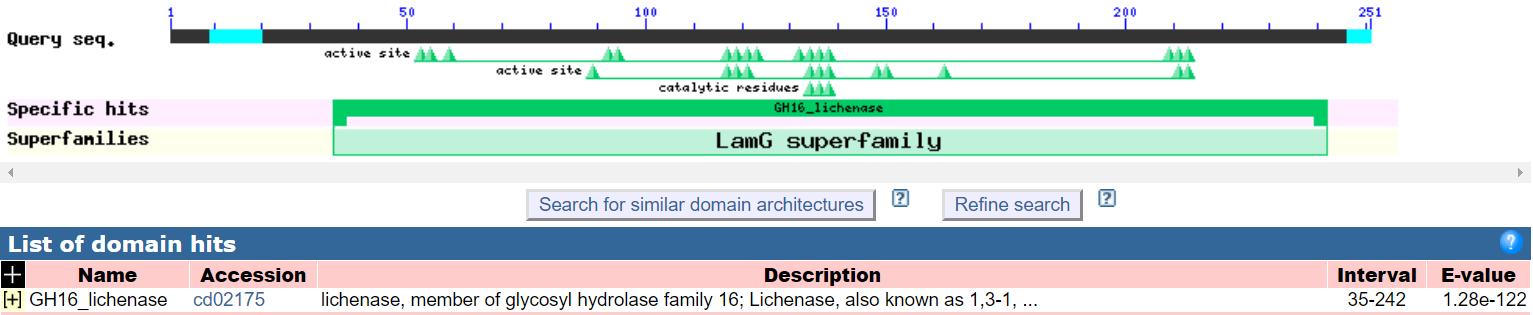


Figure S5 Domain analysis of Bgl

Supplement: Supplementary file 5 — Additional file 5: Figure S5. Domain analysis of Bgl [file 12896_2019_593_MOESM5_ESM.docx]

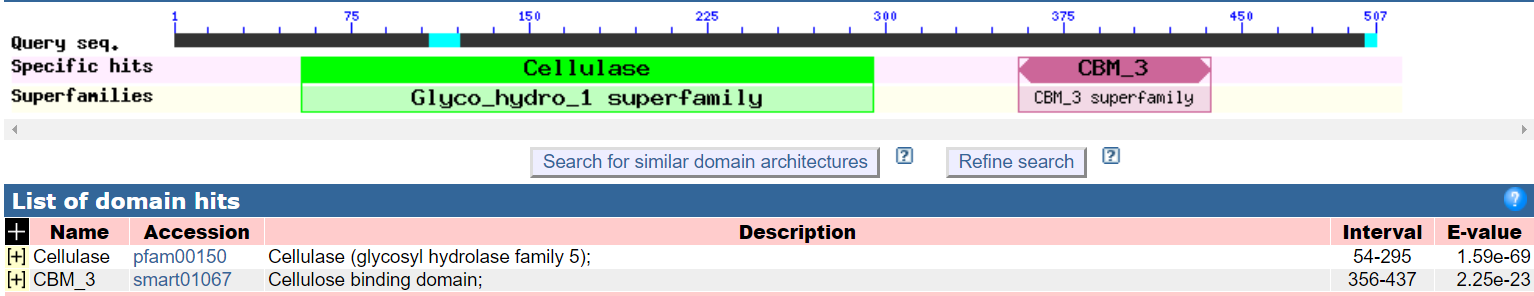
**Supplementary 6**

Figure S6 Domain analysis of Egl

Supplement: Supplementary file 6 — Additional file 6: Figure S6. Domain analysis of Egl [file 12896_2019_593_MOESM6_ESM.docx]
