# Supplementary material for: Screening of cellulolytic bacteria from rotten wood of Qinling (China) for biomass degradation and cloning of cellulases from Bacillus methylotrophicus
Source: BMC Biotechnol. 2020 Jan 7;20:2. doi: 10.1186/s12896-019-0593-8 (PMC6947901; doi:10.1186/s12896-019-0593-8)
Supplement: Supplementary file 7 — Additional file 7: Figure S7. Amino acid sequence alignments for Bgl and comparison with same gene in different strains. In which Bgl stand for β-glucosidase in this study; AS43.3 for β-glucosidase gene of Bacillus velezensis AS43.3 (CP003838.1) (complete gene); JTYP2 for Bacillus velezensis JTYP2 (CP020375.1), and 168 for Bacillus subtlis 168 (AL009126.3) [file 12896_2019_593_MOESM7_ESM.docx]

**Supplementary 7**

**
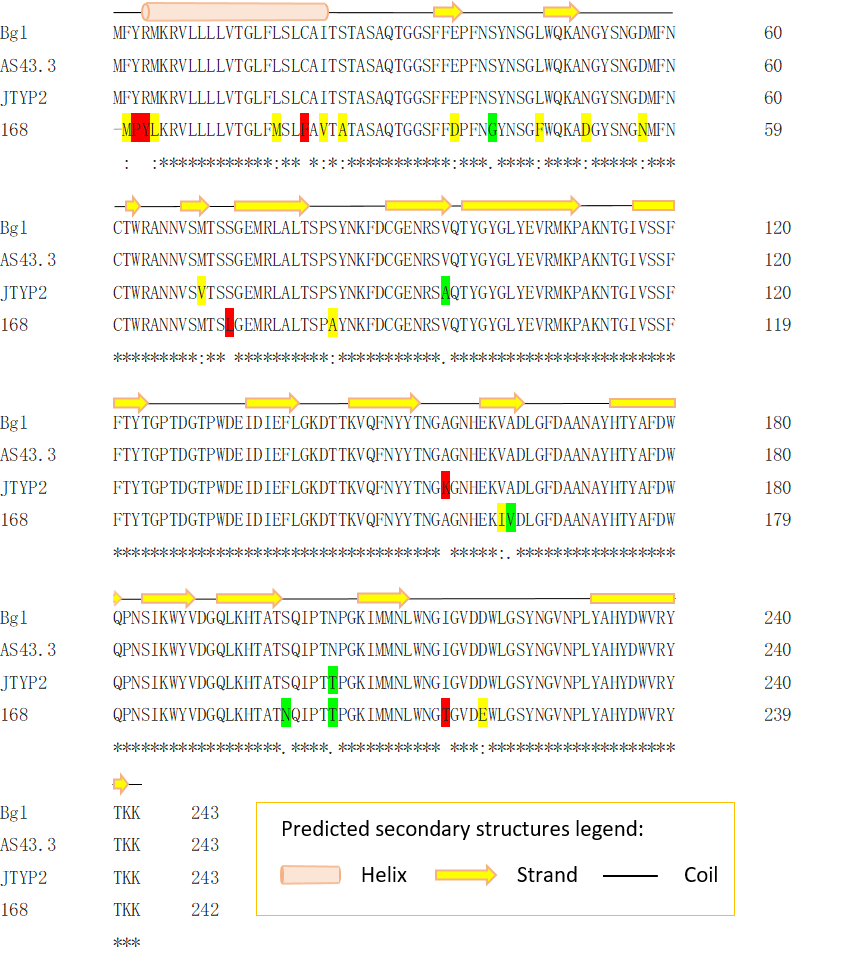
**

Figure S7 Amino acid sequence alignments for Bgl and comparison with same gene in different strains. In which Bgl stand for β-glucosidase in this study; AS43.3 for β-glucosidase gene of Bacillus velezensis AS43.3 (CP003838.1) (complete gene); JTYP2 for Bacillus velezensis JTYP2 (CP020375.1), and 168 for Bacillus subtlis 168 (AL009126.3).

Protein secondary structure of Bgl was predicted by PSIPRED. Helix, strand and coil was showed on top of aa sequence.
