# Supplementary material for: Screening of cellulolytic bacteria from rotten wood of Qinling (China) for biomass degradation and cloning of cellulases from Bacillus methylotrophicus
Source: BMC Biotechnol. 2020 Jan 7;20:2. doi: 10.1186/s12896-019-0593-8 (PMC6947901; doi:10.1186/s12896-019-0593-8)
Supplement: Supplementary file 8 — Additional file 8: Figure S8. Amino acid sequence alignments for Egl and comparison with same gene in different strains. In which Egl stand for endoglucanase in this study; JTYP2 for endoglucanase gene of Bacillus velezensis JTYP2 (CP020375.1) (complete gene); AS43.3 for Bacillus velezensis AS43.3 (CP003838.1), and 168 for Bacillus subtlis 168 (AL009126.3) [file 12896_2019_593_MOESM8_ESM.docx]

**Supplementary 8**

**
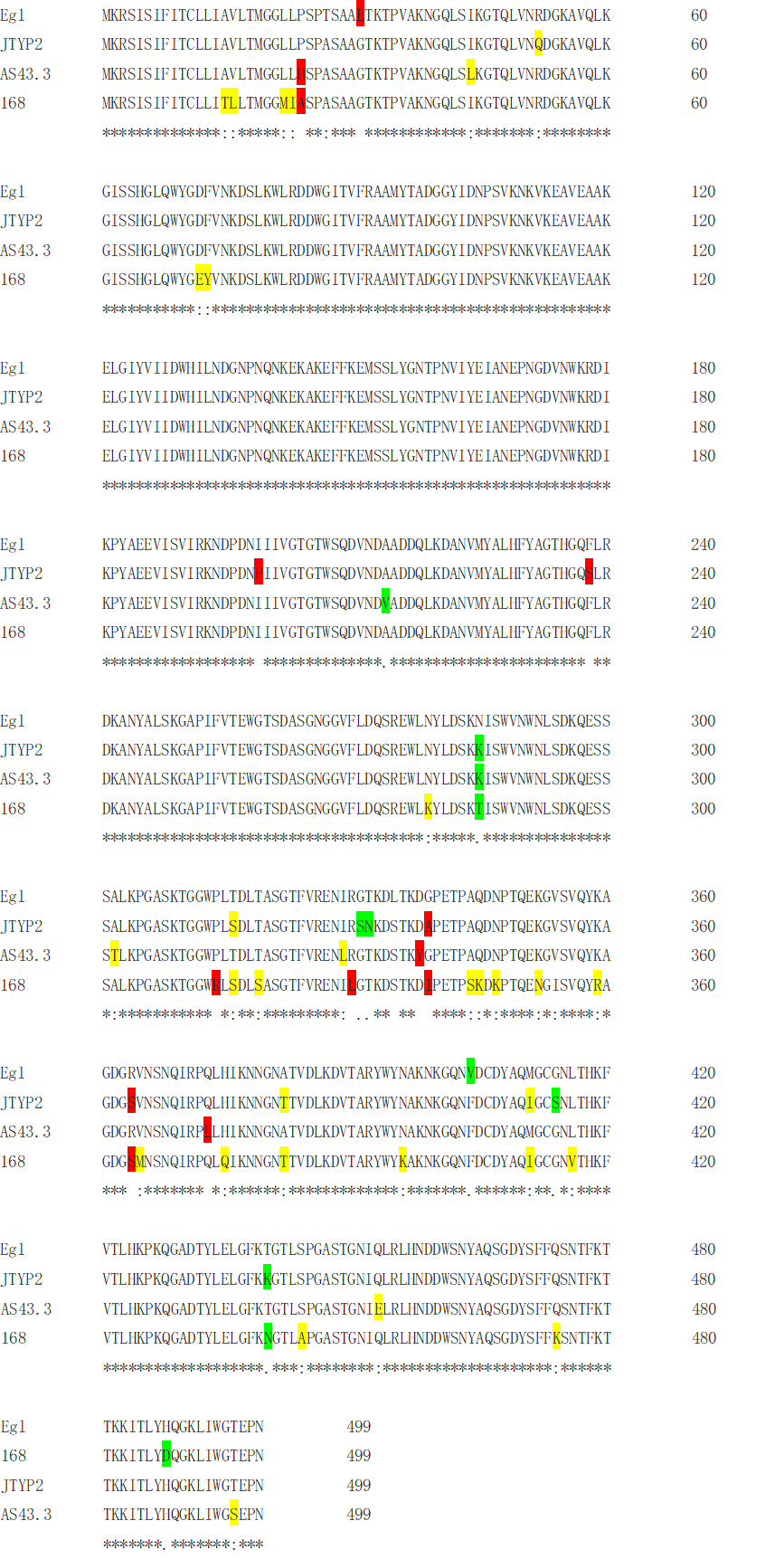
**

Figure S8 Amino acid sequence alignments for Egl and comparison with same gene in different strains. In which Egl stand for endoglucanase in this study; JTYP2 for endoglucanase gene of Bacillus velezensis JTYP2 (CP020375.1) (complete gene); AS43.3 for Bacillus velezensis AS43.3 (CP003838.1), and 168 for Bacillus subtlis 168 (AL009126.3).

Protein secondary structure of Egl was predicted by PSIPRED. Helix, strand and coil was showed on top of aa sequence.
