# Supplementary material for: Screening of cellulolytic bacteria from rotten wood of Qinling (China) for biomass degradation and cloning of cellulases from Bacillus methylotrophicus
Source: BMC Biotechnol. 2020 Jan 7;20:2. doi: 10.1186/s12896-019-0593-8 (PMC6947901; doi:10.1186/s12896-019-0593-8)
Supplement: Supplementary file 9 — Additional file 9. Accession Numbers [file 12896_2019_593_MOESM9_ESM.docx]

**Supplementary 9**

**Accession Numbers**

strain 1AJ1: MG062799, strain 1AJ2: MG062800, strain 1AJ3: MG062801, strain 1AJ4: MG062802, strain 1BJ1: MG062803, strain 1BJ2: MG062804, strain 1BJ3: MG062805, strain 1BJ4: MG062806, strain 1BJ6: MG062808, strain 1BJ7: MG062809, strain 1BJ8: MG062810, strain 1BJ9: MG062811, strain 1CJ1: MG062812, strain 1CJ2: MG062813, strain 1CJ3: MG062814, strain 1CJ4: MG062815, strain 1CJ5: MG062816, strain 1CJ6: MG062817, strain 1CJ7: MG062818, strain 1CY1: MG062819, strain 1EJ1: MG062820, strain 1EJ4: MG062821, strain 1EJ6: MG062823, strain 1EJ7: MG062824, strain 1EY1: MG062825, strain 1EY8: MG062826, strain 3AJ1: MG062827, strain 3AJ4: MG062828, strain 3AJ5: MG062829, strain 3AJ7: MG062830, strain 3BJ2: MG062831, strain 3BJ3: MG062832, strain 3BJ4: MG062833, strain 3BJ5: MG062834, strain 3BJ6: MG062835, strain 3BJ7: MG062836, strain 3BJ8: MG062837, strain 3BJ9: MG062838, strain 3CJ6: MG062839, strain 3CJ8: MG062840, strain 3CJ9: MG062841, strain 3DJ1: MG062842, strain 3DJ2: MG062843, strain 3DJ6: MG062844, strain 3EJ1: MG062845, strain 3EJ2: MG062846, strain 3EJ3: MG062847, strain 3EJ4: MG062848, strain 3EJ5: MG062849, strain 3EJ6: MG062850, strain 3EJ7: MG062851, strain 3EJ8: MG062852, strain 3EY1: MG062853, strain 3EY3: MG062854, strain 3EY4: MG062855
